# Nitrate Feed Improves Growth and Ethanol Production of *Clostridium ljungdahlii* With CO_2_ and H_2_, but Results in Stochastic Inhibition Events

**DOI:** 10.3389/fmicb.2020.00724

**Published:** 2020-05-06

**Authors:** Christian-Marco Klask, Nicolai Kliem-Kuster, Bastian Molitor, Largus T. Angenent

**Affiliations:** ^1^Environmental Biotechnology Group, Center for Applied Geoscience, University of Tübingen, Tübingen, Germany; ^2^Max Planck Fellow Groups, Max Planck Institute for Developmental Biology, Tübingen, Germany

**Keywords:** gas fermentation, *Clostridium ljungdahlii*, acetogenic bacteria, bioreactor system, pH-regulation, nitrate

## Abstract

The pH-value in fermentation broth is a critical factor for the metabolic flux and growth behavior of acetogens. A decreasing pH level throughout time due to undissociated acetic acid accumulation is anticipated under uncontrolled pH conditions such as in bottle experiments. As a result, the impact of changes in the metabolism (e.g., due to a genetic modification) might remain unclear or even unrevealed. In contrast, pH-controlled conditions can be achieved in bioreactors. Here, we present a self-built, comparatively cheap, and user-friendly multiple-bioreactor system (MBS) consisting of six pH-controlled bioreactors at a 1-L scale. We tested the functionality of the MBS by cultivating the acetogen *Clostridium ljungdahlii* with CO_2_ and H_2_ at steady-state conditions (=chemostat). The experiments (total of 10 bioreactors) were addressing the two questions: (1) does the MBS provide replicable data for gas-fermentation experiments?; and (2) does feeding nitrate influence the product spectrum under controlled pH conditions with CO_2_ and H_2_? We applied four different periods in each experiment ranging from pH 6.0 to pH 4.5. On the one hand, our data showed high reproducibility for gas-fermentation experiments with *C. ljungdahlii* under standard cultivation conditions using the MBS. On the other hand, feeding nitrate as sole N-source improved growth by up to 62% and ethanol production by 2–3-fold. However, we observed differences in growth, and acetate and ethanol production rates between all nitrate bioreactors. We explained the different performances with a pH-buffering effect that resulted from the interplay between undissociated acetic acid production and ammonium production and because of stochastic inhibition events, which led to complete crashes at different operating times.

## Introduction

An increasing world population will likely lead to growing energy demands. To meet these demands in a sustainable way, we need to rethink the *status quo* of a fossil-based economy and transition into a renewable-based and circular economy. Furthermore, we have to mitigate the apparent climate effects of anthropogenic greenhouse gas emissions, such as carbon dioxide (CO_2_), which are caused preliminary by industry, agriculture, and transportation. Biotechnology offers potential to contribute to climate-friendly and economically feasible solutions. One promising solution is synthesis gas (syngas) fermentation with microbes (Mohammadi et al., 2011). For syngas fermentation, mixtures of the gases CO_2_, hydrogen (H_2_), and carbon monoxide (CO) are converted into products, such as acetate and ethanol, by acetogenic bacteria (Dürre, 2017). This process provides a promising way to produce chemicals and biofuels with a reduced CO_2_-footprint (Latif et al., 2014; Molitor et al., 2017; Phillips et al., 2017).

In recent years, the company LanzaTech (Skokie, IL, United States) demonstrated that ethanol production from syngas with the acetogen *Clostridium autoethanogenum* is possible at commercial scale, which further indicates the potential of this platform. While the LanzaTech technology is based on proprietary strains of *C. autoethanogenum*, in academic research the most frequently studied acetogen is the closely related microbe *Clostridium ljungdahlii*. Both microbes produce acetic acid, ethanol, and some 2,3-butanediol from gaseous substrates (Tanner et al., 1993; Abrini et al., 1994; Köpke et al., 2010; Brown et al., 2014).

Different strategies are employed to optimize *C. autoethanogenum* and *C. ljungdahlii* for biotechnology. On the one hand, genetic engineering is used to generate modified strains that produce butyrate (Köpke et al., 2010), butanol (Köpke and Liew, 2012; Ueki et al., 2014), acetone, and isopropanol (Bengelsdorf et al., 2016; Köpke et al., 2016). In academic research, the physiological characterization of these genetically engineered strains is typically performed in batch experiments with serum bottles, which does not allow to control important process parameters such as the pH-value. On the other hand, bioprocess engineering is used to investigate and optimize the production of naturally occurring products, such as ethanol, in optimized bioreactor systems (Younesi et al., 2005; Mohammadi et al., 2012; Richter et al., 2013; Abubackar et al., 2015). While the impact of cultivation parameters can be investigated within one study, these studies often are difficult to compare with each other, because very different bioreactor architectures and process parameters are used (Asimakopoulos et al., 2018). Furthermore, because of the complexity, these setups are not suitable to perform preliminary experiments with genetically engineered strains. These issues can be partly overcome by utilizing commercially available bioreactor (chemostat) systems. However, these systems are costly, and therefore often not available to laboratories that do not focus on bioprocess engineering. Consequently, genetically engineered strains are typically not studied in fermentations beyond the serum bottle size, which leaves a gap between the construction of these strains and the investigation under controlled fermentation conditions.

To close this gap, we developed a cost-efficient, multiple-bioreactor system (MBS) that can be built from off-the-shelf components for a considerably smaller investment compared to the cost of commercial bioreactor systems. We give all information on purchasing the required parts, the assembly of the MBS, the process control elements (e.g., stirring, pH, temperature), and further improvement ideas. We tested our MBS with *C. ljungdahlii* and CO_2_ and H_2_ as substrate under controlled pH conditions by addressing the two questions: (1) does the MBS provide replicable data for gas-fermentation experiments?; and (2) does feeding nitrate influence the product spectrum under controlled pH conditions with CO_2_ and H_2_? In a recent study, nitrate was used as an alternative electron acceptor for *C. ljungdahlii*, while it also served as sole nitrogen source (N-source) in batch cultivations (Emerson et al., 2019). To our knowledge, this was the first study which investigated nitrate reduction by any known acetogen in detail. The co-utilization of CO_2_ and nitrate enhanced the autotrophic biomass formation with CO_2_ and H_2_ compared to standard cultivation conditions with ammonium as the sole N-source. Contrarily, ethanol production was strongly reduced under nitrate conditions with CO_2_ and H_2_. The authors discussed that nitrate reduction consumes electrons, which would be no longer available for the reduction of acetate into ethanol. At the same time, nitrate reduction led to an accumulation of ammonium. This resulted in a continuous increase of the pH from 6.0 to 8.0 during the batch cultivations in serum bottles (Emerson et al., 2019), which would intrinsically prevent ethanol production, because ethanol production is most likely triggered by a low pH (Mock et al., 2015; Richter et al., 2016). It is well-known that growth of acetogens, such as *C. ljungdahlii*, is highly dependent on the pH (Drake et al., 2008). Since their main fermentation product is acetate, which acts (in the form of the undissociated acetic acid) as a weak acid, a missing pH control, such as in serum bottles, intrinsically lowers the pH of the medium during growth. In contrast, the pH can be controlled in bioreactors such as in our MBS.

## Materials and Methods

### Microbial Strains and Medium Composition

Wild type *C. ljungdahlii* PETC (DSM 13528) was obtained from the DSMZ (Braunschweig, Germany). Generally, pre-cultures were grown heterotrophically at 37°C (IN260 stand incubator, Memmert, Germany) in 100 mL serum bottles with 50 mL of standard PETC medium containing (per liter): 0.5 g yeast extract; 1.0 g NH_4_Cl; 0.1 g KCl; 0.2 g MgSO_4_⋅7 H_2_O; 0.8 g NaCl; 0.1 g KH_2_PO_4_; 0.02 g CaCl_2_⋅2 H_2_O; 4 mL resazurin-solution (0.025 vol%); 10 ml trace element solution (TE, 100×); 10 mL Wolfe’s vitamin solution (100×); 10 mL reducing agent (100×); and 20 mL of fructose/2-(*N*-morpholino)ethanesulfonic acid (MES) solution (50×). Vitamins, reducing agent, and fructose/MES solution were added after autoclaving under sterile conditions. TE was prepared as 100x stock solution containing (per liter): 2 g nitrilotriacetic acid (NTA); 1 g MnSO_4_⋅H_2_O; 0.8 g Fe(SO_4_)_2_(NH_4_Cl)_2_⋅6 H_2_O; 0.2 g CoCl_2_⋅6 H_2_O; 0.0002 g ZnSO_4_⋅7 H_2_O; 0.2 g CuCl_2_⋅2 H_2_O; 0.02 g NiCl_2_⋅6 H_2_O; 0.02 g Na_2_MoO_4_⋅2 H_2_O; 0.02 g Na_2_SeO_4_; and 0.02 g Na_2_WO_4_. The pH of the TE was adjusted to 6.0 after adding NTA. The solution was autoclaved and stored at 4°C. Wolfe’s vitamin solution was prepared aerobically containing (per liter): 2 mg biotin; 2 mg folic acid; 10 mg pyridoxine-hydrochloride; 5 mg thiamin-HCl; 5 mg riboflavin; 5 mg nicotinic acid; 5 mg calcium pantothenate; 5 mg *p*-aminobenzoic acid; 5 mg lipoic acid; and 0.1 mg cobalamin. The vitamin solution was sterilized using a sterile filter (0.2 μm), sparged with N_2_ through a sterile filter, and stored at 4°C. The 50x fructose/MES solution contained (per 100 mL): 25 g fructose; and 10 g MES. The pH was adjusted to 6.0 by adding KOH. The solution was sterilized, sparged with N_2_ through a sterile filter, and stored at room temperature. The reducing agent was prepared under 100% N_2_ in a glove box (UniLab Pro Eco, MBraun, Germany) and contained (per 100 mL): 0.9 g NaOH; 4 g cysteine-HCl; and 2.17 g/L Na_2_S (60 weight%). Anaerobic water was used for the preparation of the reducing agent. The reducing agent was autoclaved and stored at 4°C.

For all bioreactor experiments, the standard PETC medium for the initial batch phase was supplemented with 0.5 g L^–1^ yeast extract and autoclaved inside the bioreactor vessel with an open off-gas line to enable pressure balance. The autoclaved bioreactors were slowly cooled down at room temperature overnight with an attached sterile filter at the off-gas line. After transferring each bioreactor to the MBS frame, the medium was continuously sparged with a sterile gas mixture of CO_2_ and H_2_ (20:80 vol%). After 1 h, vitamins and reducing agent were added through the sampling port. N_2_ gas was applied through a sterile filter to flush the sampling port after each addition of media components. Subsequently, each bioreactor was inoculated with 5 mL of an exponential heterotrophically grown PETC culture (OD_600_ 0.5–0.8). All feed bottles for continuous mode containing 4 L of PETC medium with additions, as described below, were autoclaved and stored overnight with an attached sterile filter on the off-gas line. The bottles were sparged with N_2_ for 2 h through a sterile filter. Vitamins and reducing agents were added under sterile conditions. A gas bag with N_2_ gas was attached with a sterile filter to balance the pressure in the feed bottle during the bioreactor run. Standard PETC medium for continuous mode did not contain yeast extract and was adjusted to the respective pH of the period. One feed bottle was simultaneously used to provide medium for three bioreactors of the same triplicate. For all nitrate experiments we replaced NH_4_Cl, with the equivalent amount of nitrogen as NaNO_3_ (18.7 mM) in the feed medium.

## Bioreactor Setup and Standard Operating Conditions

**FIGURE 1:**
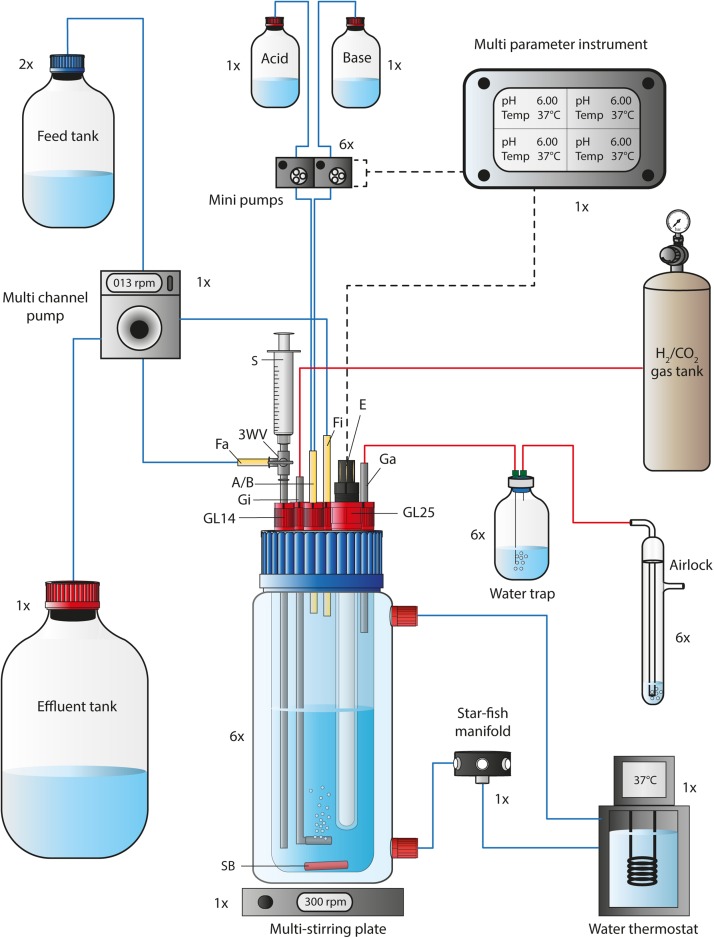
Flow chart of a single bioreactor operated in the MBS. The 1-L bioreactor vessel consisted of a double-walled glass vessel and a customized lid, while it was placed on a multi-stirring plate with up to six bioreactors. The bioreactor temperature was maintained through a water circulation unit at 37°C. The autoclavable lid offered connections for 5x GL14 and 1x GL25. A set of stainless-steel tubing was used for the gas-in/-out lines and for the medium feed-out line. The three-way valve at the medium feed-out line was required for sampling using a 5-mL syringe. The pH and bioreactor medium temperature was tracked *via* a pH/pt1000-electrode that was connected to a multi-parameter instrument. The multi-parameter instrument controlled and triggered two mini pumps (for base and acid) at programmable conditions. For continuous mode, the feed medium to each bioreactor was pumped *via* a single multi-channel pump from the feed tank into the bioreactor. The same pump was used to transfer the effluent from each bioreactor into the effluent tank. Sterile CO_2_ and H_2_ gas (20:80 vol-%) was sparged into the system through stainless-steel tubing with an attached sparger. The gas-out line was connected to a 100-mL serum bottle to serve as a water trap before the outgoing gas passed an airlock. The 1×, 2×, and 6× next to each unit in the figure describe the quantity, which is required to operate six bioreactors simultaneously. A/B, Acid and/or base feed line; E, pH/pt1000 electrode; Fa, medium feed-out line; Fi, medium feed-in line; Ga, gas-out line; Gi, gas-in line; GL14, screw joint connection size 14; GL25, screw joint connection size 25; rpm, revolutions per minute; SB, stirring bar; 3WV, three-way valve. Blue lines indicate liquid transfer, red lines contain gas, and dotted black lines provide electric power or signals.

**FIGURE 2:**
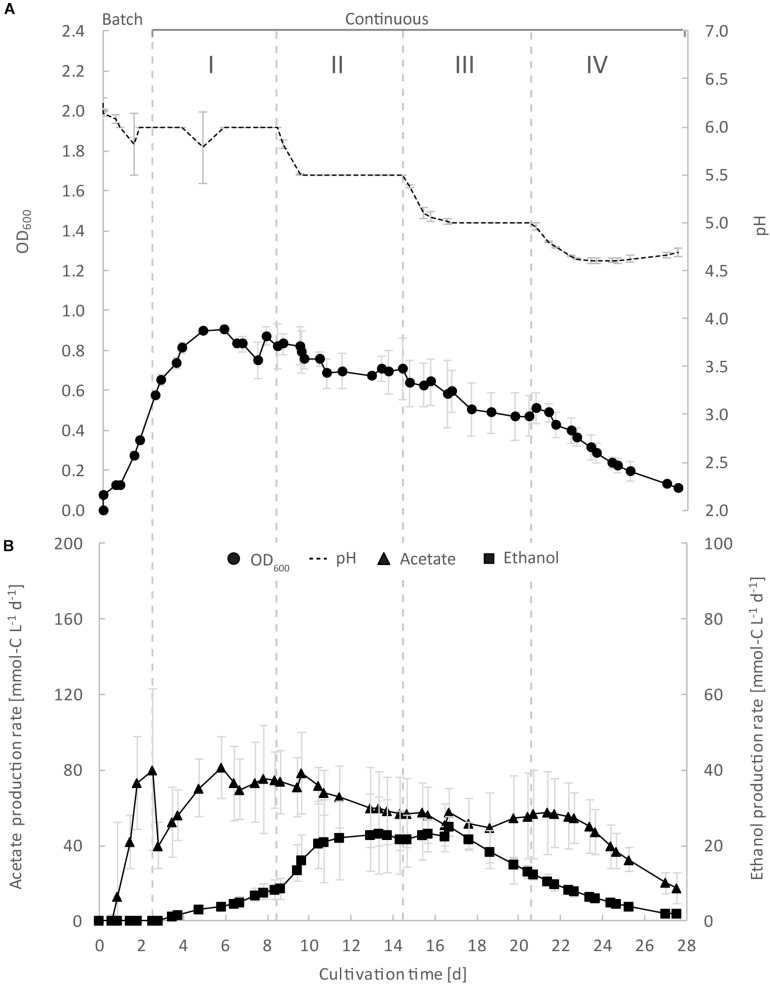
Continuous gas fermentation of *C. ljungdahlii* with CO_2_ and H_2_ in standard PETC medium at different periods in a priliminary experiment (bioreactor 1/2/3). Mean values of triplicates with standard deviation (*n* = 3) for pH and OD_600_ **(A)**, and for acetate and ethanol production rates in mmol-C L^–1^ d^–1^ **(B)**. Standard PETC medium containing 18.7 mM ammonium chloride as sole N-source was used. The horizontal dotted lines indicate the continuous process in which medium of different pH was fed to each bioreactor. Period: I, pH = 6.0; II, pH = 5.5, III, pH = 5.0; and IV, pH = 4.5.

**FIGURE 3:**
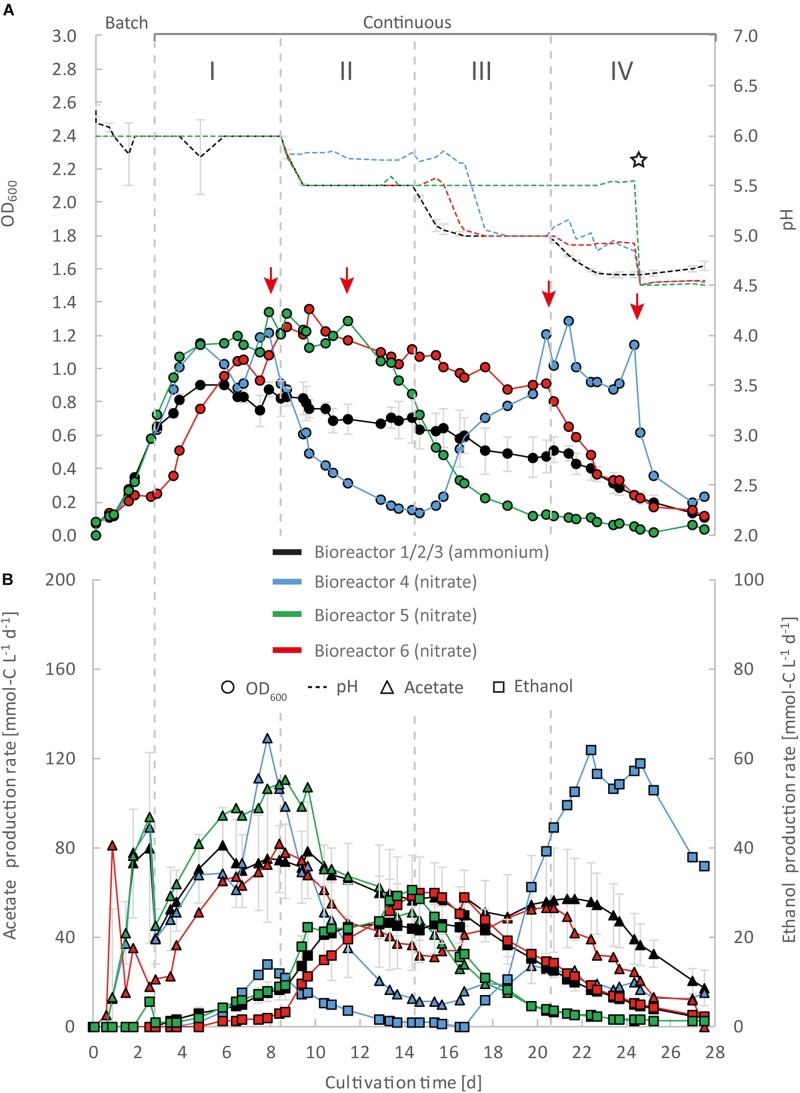
Impact of nitrate as an alternative N-source on continuous gas fermentation of *C. ljungdahlii* using CO_2_ and H_2_ at different periods with a medium feed rate of 0.10 mL min^–1^ (experiment 1). Single values for pH and OD_600_ **(A)**, and for acetate and ethanol production rates in mmol-C L^–1^ d^–1^ **(B)**. The bioreactors with nitrate feed were grown in ammonium-free PETC medium supplemented with 18.7 mM Na-nitrate. The horizontal dotted lines indicate the continuous process in which medium of different pH was fed to each bioreactor. The red arrows indicate the crash in OD_600_ of each bioreactor with nitrate feed at different time points. The star symbol describes the time point were the pH was lowered manually by adding HCl to the system until a pH of 4.5 was reached. Period: I, pH = 6.0; II, pH = 5.5, III, pH = 5.0; and IV, pH = 4.5.

**FIGURE 4:**
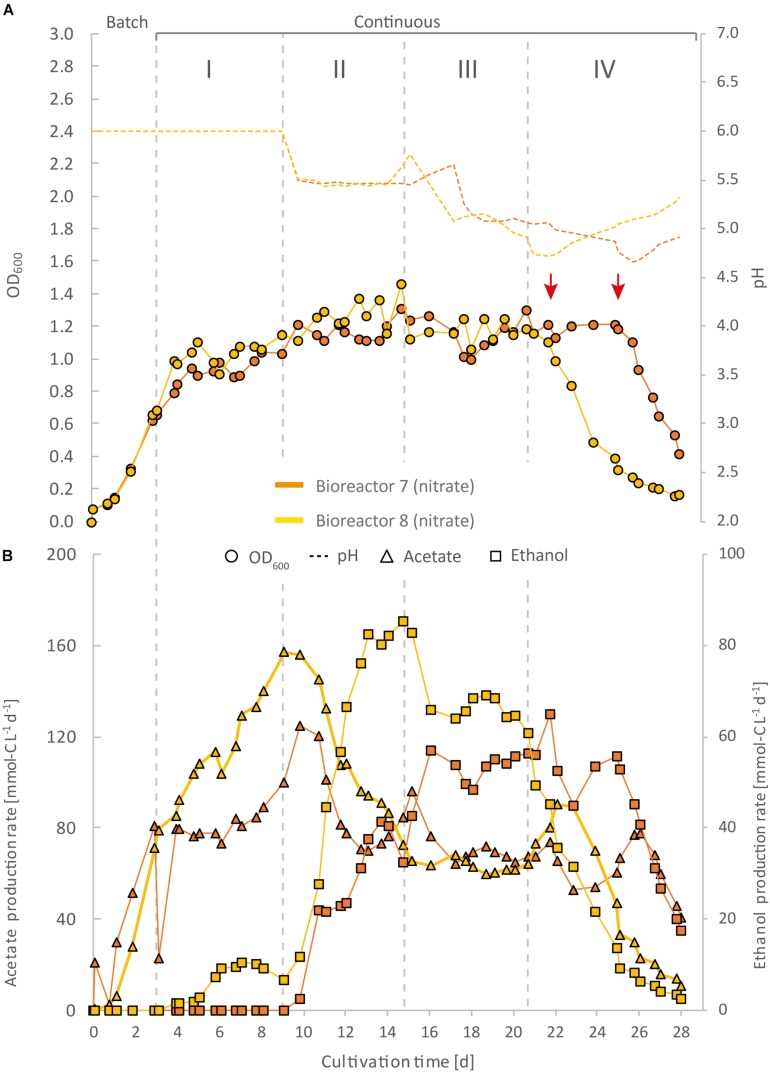
Impact of nitrate as an alternative N-source on continuous gas fermentation of *C. ljungdahlii* using CO_2_ and H_2_ at different periods with a medium feed rate of 0.19 mL min^–1^ (experiment 2). Single values for pH and OD_600_ **(A)**, and for acetate and ethanol production rates in mmol-C L^–1^ d^–1^ **(B)**. The bioreactors were grown in ammonium-free PETC medium supplemented with 18.7 mM Na-nitrate. The horizontal dotted lines indicate the continuous process in which medium of different pH was fed to each bioreactor. The red arrows indicate the crash in OD_600_ of each bioreactor at different time points. Period: I, pH = 6.0; II, pH = 5.5, III, pH = 5.0; and IV, pH = 4.5.

**FIGURE 5:**
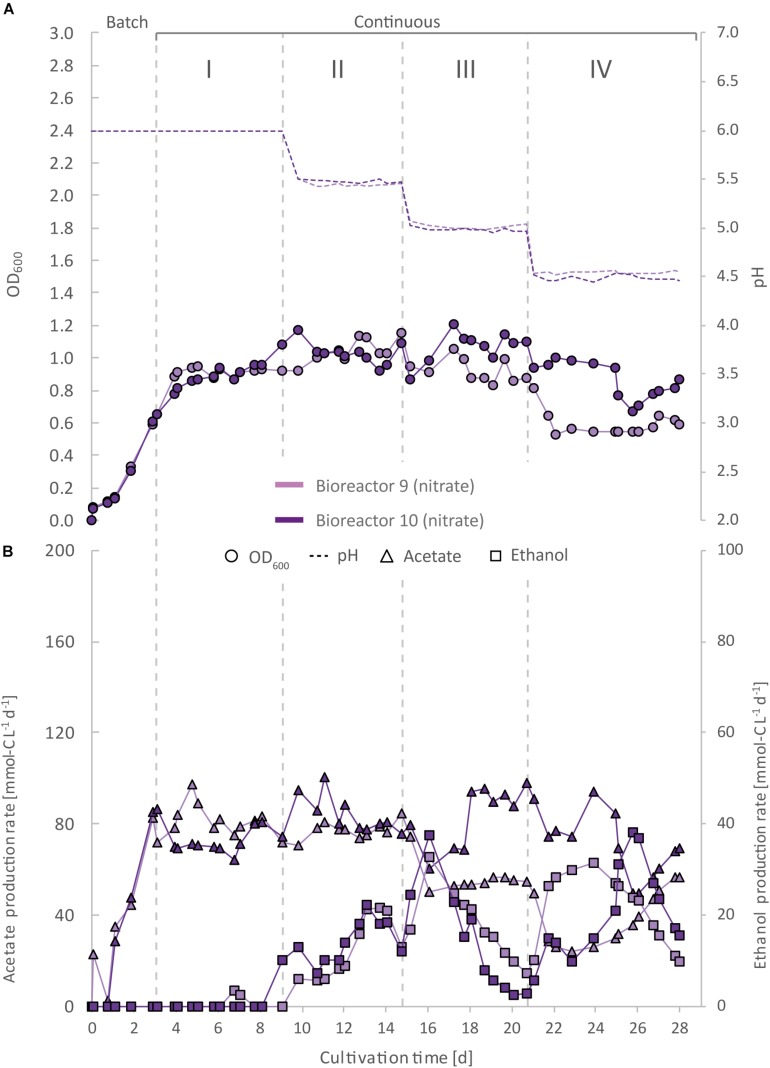
Impact of nitrate as an alternative N-source on continuous gas fermentation of *C. ljungdahlii* using CO_2_ and H_2_ at different periods with a medium feed rate of 0.19 mL min^–1^ (experiment 3). Single values for pH and OD_600_ **(A)**, and for acetate and ethanol production rates in mmol-C L^–1^ d^–1^ **(B)**. The bioreactors were grown in ammonium-free PETC medium supplemented with 18.7 mM Na-nitrate. The horizontal dotted lines indicate the continuous process in which medium of different pH was fed to each bioreactor. Period: I, pH = 6.0; II, pH = 5.5, III, pH = 5.0; and IV, pH = 4.5.

Six 1 L self-built bioreactors (Figure 1, Supplementary Data Sheet S1, Supplementary Figures S1–S3, Supplementary Results S1, and Supplementary Table S1 in Supplementary Data Sheet S2) with a working volume of 0.5 L (1-L double walled jacketed GLS 80 bottle, Duran, Germany) were operated simultaneously for two experimental bioreactor runs, while four of these bioreactors were operated simultaneously for two additional experimental bioreactor runs, with a total of 10 bioreactors (Figures 2–5). The cultivation temperature was 37°C and the agitation was set to 300 rpm. The gas flow rate was adjusted to 30 mL min^–1^ *prior* to inoculation. To establish microbial growth in the MBS after one inoculation event for each bioreactor, we operated the MBS in batch mode for 3–4 days before switching to continuous mode. The pH was set to 6.0 during the batch mode and the first 6 days (Period I) in continuous mode. Subsequently, the pH setting was lowered stepwise in 6 days to a pH of 5.5, 5.0, and 4.5 (Period II-IV). The pH of the feed medium was adjusted to the anticipated pH of each period. In our preliminary experiment (bioreactor 1/2/3) and the first nitrate experiment (bioreactor 4/5/6) (Figures 2, 3), we did not use the acid feed to actively adjust the pH within the bioreactor. Instead, we let the pH decrease to the set value by the microbial production of undissociated acetic acid to avoid a pH shock. This took approximately one to 2 days of each 6-day period. For the preliminary experiment and the first nitrate experiment we chose a medium feed rate of 0.10 mL min^–1^, which resulted in a 3.5-day hydraulic retention time (HRT), and which represents 1.7 HRT periods within each period of 6 days. In our second and third nitrate experiment, the medium feed rate was 0.19 mL min^–1^. This was equal to a 2-day HRT and resulted in 3.2 HRT periods within each pH period. We only used base feed to maintain the pH in the second nitrate experiment (bioreactor 7 and 8) (Figure 4), while base and acid feed was actively applied for the third nitrate experiment (bioreactor 9 and 10) (Figure 5) to immediately adjust the pH of the bioreactor to the anticipated pH-value of each period. We used 2 M KOH and 2 M HCl in our experiments.

### Sampling and Analyses

Bioreactors were sampled once or twice per day. A pre-sample of 3 mL of cell suspension was discarded, before taking a 2 mL sample (main sample) during batch mode. For sampling in continuous mode, the multi-channel pump (Masterflex L/S pump equipped with a Multichannel Cartridge Pump Head and twelve catridges, Cole Parmer, Germany) was switched off during the sampling procedure. Cell growth was monitored by measuring the optical density at 600 nm (OD_600_) (Nanophotometer NP80, Implen, Germany). For OD_600_-values larger than 0.5, dilutions with 100 mM phosphate-buffered saline (PBS) at pH 7.4 were prepared. Nitrate and nitrite concentrations were qualitatively monitored using test stripes (Quantofix nitrate/nitrite, Macherey-Nagel, Germany). A correlation between cellular dry weight (CDW) and OD_600_ was calculated by harvesting 50 mL of culture sample from every bioreactor, centrifugation of the samples at 3428 relative centrifugal force (rcf) (Eppendorf centrifuge 5920R) for 12 min at room temperature (RT) and, subsequently, drying the pellet at 65°C for 3 days. The CDW for an OD_600_ of 1 was determined to be 0.24 g L^–1^ for cultures grown in PETC medium with ammonium and 0.29 g L^–1^ for cultures grown in PETC medium with nitrate as sole nitrogen source, respectively.

Acetate and ethanol concentrations were analyzed *via* a high-pressure liquid chromatography (HPLC) (LC20, Shimadzu, Japan) system that was equipped with an Aminex HPX-87H column and operated with 5 mM sulfuric acid as eluent. The flow was 0.6 mL min^–1^ (LC-20AD). The oven temperature was 65°C (CTO-20AC). The sample rack of the HPLC was constantly cooled to 15°C in the autosampler unit (SIL-20AC_HT_). For HPLC sample preparation, all culture samples were centrifuged for 3 min at 15871 rcf (Centrifuge 5424, Eppendorf, Germany) in 1.5 mL reaction tubes. 750 μl of the supernatant was transferred into clean reaction tubes and stored at −20°C until use. Frozen samples were thawed at 30°C and 250 revolutions per minute (rpm) for 10 min (Thermomixer C, Eppendorf, Germany). The samples were centrifuged again and 500 μl of the supernatant was transferred into short thread HPLC/GC vials (glass vial ND9, VWR, Germany) and sealed with short screw caps, which contained rubber septa (6 mm for ND9, VWR, Germany). New standards for acetate and ethanol were prepared for every analysis. All HPLC samples were randomized.

## Results

### Operating the MBS for Replicable Gas Fermentation Experiments

We based our experiments in this study on a versatile self-built multiple-bioreactor system (MBS). The MBS (Figure 1, Supplementary Data Sheet S1, Supplementary Figures S1–S3, Supplementary Table S1, and Supplementary Results S1 in Supplementary Data Sheet S2) was designed to either perform heterotrophic or autotrophic cultivation experiments in batch or continuous mode. The MBS can be used to operate up to six bioreactors simultaneously, each individually at different pH conditions or, if necessary, with different feed medium. The MBS platform might be especially interesting for cost-effective research in academia.

To show high comparability and reproducibility of our MBS, as a preliminary experiment (control), we grew *C. ljungdahlii* simultaneously as triplicates in standard PETC medium with CO_2_ and H_2_ (ammonium, bioreactors 1/2/3) (Figure 2 and Supplementary Figure S4 in Supplementary Data Sheet S2). We observed that growth was similar in the triplicate bioreactors during the cultivation of 27.5 days. During the initial batch mode, the average OD_600_ increased to 0.58 ± 0.01 (Figure 2A). After switching to continuous mode, the average OD_600_ increased further to values of 0.82 ± 0.04 during Period I. For Periods II, III, and IV, the average OD_600_ for the bioreactors constantly decreased to values of 0.69 ± 0.01, 0.51 ± 0.05, and 0.18 ± 0.06 (Figure 2A and Table 1). As expected, the pH of each bioreactor was decreasing during all periods by microbial acetate production. The simultaneous and constant decrease of OD_600_ indicated reduced growth rates of *C. ljungdahlii* at a lower pH level in our system. In batch mode, the acetate production rates increased with increasing OD_600_, but then considerably dropped after switching to continuous mode (Figure 2A). The acetate production rates increased again to the highest measured average value of 73.1 ± 2.1 mmol-C L^–1^ d^–1^ for Period I (Figure 2B). The acetate production rates decreased to average values of 60.1 ± 3.4 mmol-C L^–1^ d^–1^, and 53.7 ± 3.3 mmol-C L^–1^ d^–1^ for Periods II and III, respectively. For Period IV, the acetate production rate had only an average value of 29.2 ± 10.0 mmol-C L^–1^ d^–1^ (Figure 2B). Ethanol production rates were negligible during batch mode, but slowly increased after switching to continuous mode. The highest ethanol production rates were observed for Period II with average values of 22.5 ± 0.5 mmol-C L^–1^ d^–1^. During the Periods III and IV, the ethanol production rates kept decreasing to average values of 18.7 ± 4.8 mmol-C L^–1^ d^–1^ and 3.4 ± 1.4 mmol-C L^–1^ d^–1^, respectively (Figure 2B). The results of our preliminary experiment showed high reproducibility with small standard deviations for all tested parameters using the MBS, which creates an environment to investigate the impact of different cultivation parameters simultaneously in a single system providing statistically relevant fermentation data.

**TABLE 1:** Average values for OD_600_ and acetate/ethanol production rates during the continuous fermentation of *C. ljungdahlii* with CO_2_ and H_2_ at four different pH conditions in standard PETC medium using the MBS (priliminary experiment, control).

Operating conditions	OD_600_^1^	Acetate production rate [mmol-C L^–^^1^ d^–^^1^]^1^	Ethanol production rate [mmol-C L^–^^1^ d^–^^1^]^1^	Ratio_Et/Ac_^2^
Period I (pH 6.0)	0.82 ± 0.04	73.1 ± 2.1	6.4 ± 1.6	0.1
Period II (pH 5.5)	0.69 ± 0.01	60.1 ± 3.4	22.5 ± 0.5	0.4
Period III (pH 5.0)	0.51 ± 0.05	53.7 ± 3.3	18.7 ± 4.8	0.3
Period IV (pH 4.5)	0.18 ± 0.06	29.2 ± 10.0	3.4 ± 1.4	0.1

*^1^Values for the bioreactors with ammonium feed (*n* = 3) are given as the average (±standard deviation) from three bioreactors for the last 5 data points of every period. ^2^Et, Ethanol; Ac, Acetate.*

### Feeding Nitrate to *C. ljungdahlii* in Continuous Operating Bioreactors With H_2_ and CO_2_

For three main experiments with operating periods of 27.5 days (experiment 1), and 28 days (experiments 2 and 3), we investigated the impact of nitrate as an alternative N-source on growth and the production of ethanol from CO_2_ and H_2_ (Figures 3–5). For these experiments, the bioreactors (experiment 1, bioreactor 4/5/6; experiment 2, bioreactor 7/8; experiment 3, bioreactor 9/10) were fed with PETC medium containing nitrate instead of ammonium at an equivalent molar amount of nitrogen (=18.7 mM). We found an increasing pH due to ammonium production in preliminary bottle experiments in nitrate-containing PETC medium (Supplementary Figure S5 in Supplementary Data Sheet S2). A pH increase was also observed in the nitrate bottle experiments of Emerson et al. (2019). Despite the pH-control in our experiments, all bioreactors with nitrate feed showed remarkable differences in growth, pH, acetate production, and ethanol production rates. Therefore, we report individual data for each bioreactor and highlight lowest and highest values (Tables 2, 3). We use the data of the preliminary experiment (ammonium, bioreactor 1/2/3) as the control in which ammonium served as the sole N-source (Figure 3). Unexpectedly, we observed a pH-buffering effect for experiment 1 (bioreactor 4/5/6) and experiment 2 (bioreactor 7/8) with nitrate feed during the fermentation (Figures 3A, 4A). This was most likely due to an interplay between the produced acetate and ammonium by the microbes. Overall, the pH was slowly decreasing in these bioreactors with nitrate feed (Figures 3–5), and we did not measure increasing pH values. To study this effect further, we actively reduced the pH in every pH period to the anticipated pH-value by feeding acid for experiment 3 (bioreactor 9/10).

**TABLE 2:** Highest observed values for OD_600_ and acetate/ethanol production rates at specific pH during continuous fermentation of *C. ljungdahlii* with CO_2_ and H_2_ in nitrate-containing medium with a feed rate of 0.10 mL min**^–^**^1^.

Highest value for	Control^1,2^	Experiment 1^2^		
	Bioreactor 1-3 (ammonium)	Bioreactor 4 (nitrate)	Bioreactor 5 (nitrate)	Bioreactor 6 (nitrate)
OD_600_	0.90 ± 0.02 (pH 6.0)	1.29(*pH*5.2)	1.36(*pH*5.5)	1.34(*pH*6.0)
Acetate production rate [mmol-C L^–1^ d^–1^]	81.4 ± 3.0 (pH 6.0)	128.8(*pH*6.0)	81.5(*pH*6.0)	110.6(*pH*5.8)
Ethanol production rate [mmol-C L^1^ d^–1^]	25.0 ± 2.7 (pH 5.0)	62.0(*pH*5.0)	29.9(*pH*5.0)	30.6(*pH*5.5)
Ratio_Et/Ac_^3^	0.4 (pH 5.5)	4.2(*pH*4.5)	1.0(*pH*5.6)	0.6(*pH*5.5)

*^1^Values for the bioreactors with ammonium feed (*n* = 3) are given as the average (±standard deviation) from three bioreactors for the last 5 data points. ^2^ Only base was fed to maintain the pH, while a pH decrease was caused by microbial acetic acid production. ^3^Et, Ethanol; Ac, Acetate.*

**TABLE 3:** Highest observed values for OD_600_ and acetate/ethanol production rates at specific pH during continuous fermentation of *C. ljungdahlii* with CO_2_ and H_2_ in nitrate-containing medium with a feed rate of 0.19 mL min**^–^**^1^.

Highest value for	Experiment 2^1^		Experiment 3^2^	
	Bioreactor 7 (nitrate)	Bioreactor 8 (nitrate)	Bioreactor 9 (nitrate)	Bioreactor 10 (nitrate)
OD_600_	1.31(*pH*5.5)	1.46(*pH*5.6)	1.16(*pH*5.5)	1.21(*pH*5.0)
Acetate production rate [mmol-C L^–1^ d^–1^]	124.8(*pH*5.5)	139.2(*pH*5.5)	97.4(*pH*6.0)	100.7(*pH*5.5)
Ethanol production rate [mmol-C L^–1^ d^–1^]	65.1(*pH*5.1)	85.4(*pH*5.6)	32.6(*pH*5.0)	38.1(*pH*4.5)
Ratio_Et/Ac_^3^	1.0(*pH*4.9)	1.4(*pH*5.8)	1.2(*pH*4.6)	0.8(*pH*4.5)

*^1^Only base was fed to maintain the pH of the bioreactor, while a pH decrease was caused by microbial acetic acid production. ^2^Base and acid were fed to maintain the pH of the bioreactor. The pH was actively decreased to the feed medium pH when entering a new pH period. ^3^Et, Ethanol; Ac, Acetate.*

During the initial batch mode of all performed bioreactor experiments, growth was similar in each bioreactor and reached highest OD_600_-values between 0.5 and 0.7 after 2–3 days (Figures 2A–5A). In the first nitrate experiment, bioreactor 6 stagnated after 2 days of cultivation in batch with an OD_600_ of 0.23 (Figure 3A). However, after switching to continuous mode, all three nitrate bioreactors of experiment 1 (bioreactor 4/5/6) reached similar OD_600_-values of ∼1.2 during the end of Period I, which were 48% higher compared to the mean OD_600_-value of the control bioreactors with ammonium feed and the same medium feed rate during Period I (Figures 2A, 3A and Table 1). The highest observed OD_600_ was 1.29 on day 21 during Period IV for bioreactor 4, 1.36 on day 10 during Period II for bioreactor 5, and 1.34 on day 8 during Period I for bioreactor 6 (Figure 3 and Table 2). In comparison, the bioreactors with ammonium feed had the highest average OD_600_-value of 0.90 ± 0.02 on day 4 for Period I (Figure 2 and Table 1). The increase of biomass was also observed in our second and third nitrate experiment (Figures 4A, 5A and Table 3). When applying higher medium feed rates in these experiments, the highest OD_600_-values were increased by 29–62% compared to the ammonium bioreactors (Figures 4A, 5A and Tables 2, 3). This indicated that the increased dilution rate did not exceed the growth rate of the microbes under our conditions. The highest OD_600_-values for the bioreactors of experiments 2 and 3 were 1.31 for bioreactor 7 on day 15 during Period II, 1.46 for bioreactor 8 on day 15 during Period II, 1.16 for bioreactor 9 on day 15 during Period II, and 1.21 for bioreactor 10 on day 17 during Period III.

A noticeable difference was that the OD_600_-values were unstable for all bioreactors with nitrate feed (Figures 3A–5A). Each nitrate bioreactor showed fluctuating OD_600_-values of ±0.1 to ±0.3 during the experiment. This effect was not observed for the control bioreactors with ammonium feed, which showed continuously decreasing OD_600_-values (Figure 2A). However, the fluctuating growth of the nitrate bioreactors was interrupted, when crash events occurred at different time points during the first and second nitrate experiment (Figures 3A, 4A, red arrow). We observed these crash events in OD_600_ for bioreactor 4 on day 8 during Period I and again on day 24 during Period IV, for bioreactor 5 on day 20 during Period III, and for bioreactor 6 on day 11 at the end of Period III. The second crash event of bioreactor 4 was observed after a previous phase of recovery during Period III in which the cells grew again to an OD_600_ of 1.2 on day 20 (Figure 3A). The recovery of growth was only observed for bioreactor 4. Crash events also occurred at the higher medium feed rates in our second nitrate experiment, but at later time points of the cultivation (Figure 4A). Bioreactor 7 underwent a crash event on day 26 during Period IV, while bioreactor 8 crashed on day 21 during Period IV. It is noteworthy, that we detected nitrate and nitrite in culture samples of all nitrate bioreactors undergoing a crash event, while neither nitrate nor nitrite were detectable in actively growing or recovering bioreactors with nitrate feed (see Supplementary Data Sheet S3). This indicates a high uptake rate for nitrate by the microbes from the feed medium, and an immediate conversion of the nitrate to ammonium *via* nitrite as an intermediate. During our third nitrate experiment (bioreactor 9 and 10) (Figure 5), we neither observed crash events nor the accumulation of nitrate or nitrate in any culture sample. Nevertheless, both bioreactors 9 and 10 showed a short duration of decreasing OD_600_-values during Period IV, but their growth remained stable afterward. Despite the occurrence of crash events, we found that all nitrate bioreactors showed high OD_600_-values even at lower pH (Period II-IV (Figures 3A–5A and Tables 2, 3). In contrast, the control bioreactors, growing with ammonium feed, showed high OD_600_-values only at pH 6.0, while the OD_600_ kept constantly decreasing at lower pH (Figure 2A, Supplementary Figure S4 in Supplementary Data Sheet S2, and Table 1).

The acetate production rates of all bioreactors with nitrate feed somewhat followed the OD_600_ profile, and reached the highest values that we observed in all our experiments with a maximum value of 139 mmol-C L^–1^ d^–1^ for bioreactor 8 of experiment 2 during Period II (Figure 4B and Table 3). Overall, the acetate production rates were more stable during experiment 3, when the pH was actively decreased with an acid feed (Figure 5B). The acetate production rate considerably decreased at the time point of the OD_600_ crashes for the three bioreactors for experiment 1 and the two bioreactors for experiment 2 (Figures 3B,  4B).

Ethanol production rates were negligible during batch mode for all bioreactors with nitrate feed, and increased with decreasing pH during the different periods. After switching to continuous mode, and considerably dropped for each bioreactor that crashed (Figures 2B–5B). For our first nitrate experiment with a medium feed rate of 0.10 mL min^–1^, we observed similar ethanol production rates for bioreactor 5 and 6 compared to the control experiment with ammonium feed (Figure 4B). The highest ethanol production rates were 30 mmol-C L^–1^ d^–1^ for bioreactor 5 on day 15 during Period III and 31 mmol-C L^–1^ d^–1^ for bioreactor 6 on day 14 during Period II. While for bioreactors 5 and 6 the ethanol production rates did not recover after the crashes, for bioreactor 4 the ethanol production rate increased with increasing OD_600_ after the crash, and reached a maximum of 62 mmol-C L^–1^ d^–1^ on day 22 during Period IV. This value is ∼2.5-fold higher compared to the highest ethanol production rate observed for *C. ljungdahlii* growing with ammonium (Figure 2B and Table 2) with the same medium feed rate of 0.10 mL min^–1^. When we applied a higher medium feed rate of 0.19 ml min^–1^, we found that ethanol production was strongly enhanced for bioreactor 7 and bioreactor 8 for experiment 2 (Figure 4B and Table 3). Highest ethanol production rates were 65 mmol-C L^–1^ d^–1^ for bioreactor 7 on day 22 during Period IV and 85 mmol-C L^–1^ d^–1^ for bioreactor 8 on day 15 during Period II. On the contrary, the ethanol production rates of bioreactor 9 and bioreactor 10 for experiment 3 that operated with the same medium feed rate but with acid feed to control the pH, were lower with highest rates of 33 mmol-C L^–1^ d^–1^ for bioreactor 9 and 38 mmol-C L^–1^ d^–1^ for bioreactor 10, both on day 16 during Period III (Figure 5B and Table 3). It should be noted that acetate production rates of bioreactor 4 remained low after the recovery, which led to the highest measured ethanol/acetate ratio of ∼4.2 with CO_2_ and H_2_ in this study (Table 2). To our knowledge it is also the highest ethanol/acetate ratio for published studies with acetogens and CO_2_ and H_2_, because Mock et al. (2015) achieved a ratio of ∼1:1. In contrast, the acetate production rates of bioreactor 7, 8, 9, and 10 for experiment 2 and 3 were similar, even when we observed the high ethanol production rates in bioreactor 7 and 8 (Table 3).

We found in our first nitrate experiment with a medium feed rate of 0.10 mL min^–1^ that each bioreactor behaved differently and underwent stochastic crashes in the OD_600_ at different time points that were most likely connected to a simultaneous accumulation of nitrite (Figure 3A). One bioreactor recovered from this crash and showed increased ethanol production rates after the crash (Figure 3B). When we increased the medium feed rate to 0.19 mL min^–1^ for experiment 2, we observed again crash events at different time points (Figure 4A). Interestingly. before these crashes, these bioreactors already showed increased ethanol production rates compared to experiment 1. Furthermore, we found that crash events did not occur for experiment 3 during which we actively and immediately decreased the pH to the anticipated pH-value (Figure 5A). However, overall ethanol production rates were lower then. It is likely that the simultaneous production of acetate from acetogenesis and ammonium from nitrate reduction creates a sensitive environment for *C. ljungdahlii*, which supports growth and production rates of ethanol through a self-buffering pH effect of the cell, but with a high instability of the system, as discussed in detail below.

## Discussion

### Our MBS Resulted in Reproducible Gas-Fermentation Experiments With *C. ljungdahlii*

The MBS was successfully tested to cultivate *C. ljungdahlii* with CO_2_ and H_2_ under various pH conditions during four experiments (total of 10 bioreactors). The highly comparable growth behavior of the triplicate bioreactors under batch and continuous conditions in the preliminary experiment, using standard medium with ammonium as the N-source (control), confirm a high stability of our MBS (Figure 2A and Supplementary Figure S4A in Supplementary Data Sheet S2). We did observe minor differences for the preliminary experiment in the ethanol and acetate production rates between replicates, which were connected to the same medium feed bottle under continuous conditions (Figure 2B and Supplementary Figure S4B in Supplementary Data Sheet S2). These differences in single replicates may lead to different production rates, even in controlled bioreactors, and may result from slightly varying gassing or medium feed rates, variations in the pH control, or small but varying diffusion of oxygen into individual bioreactors. This finding clearly indicates the need for replicates during strain characterization and pre-selection in lab-scale bioreactor experiments before scaling up to larger fermentations. With our MBS, we can combine experiments at steady-state conditions for replicates, which saves time in generating statistically relevant data sets. Our future work to further optimize the MBS will target the additional integration of analytic equipment to calculate gas consumption and carbon uptake rates. We had sampled the inlet and outlet gases during all experiments, but our current setup was not adequate to obtain reliable results. Additional equipment, such as mass-flow controllers, will fill this gap and further increase the data quality during future experiments.

### Feeding Nitrate as Sole N-Source Led to Enhanced Cell Growth Even at Low pH

For our three main experiments (Figures 3–5), we tested the impact of nitrate as sole N-source on the growth and production rates of acetate and ethanol under pH-controlled conditions. It was recently demonstrated that *C. ljungdahlii* can use nitrate simultaneously for the generation of ammonium (assimilatory nitrate reduction) (Nagarajan et al., 2013), and as an alternative electron acceptor (dissimilatory nitrate reduction) (Emerson et al., 2019). This resulted in enhanced cell growth with sugars or CO_2_ and H_2_ in bottle experiments (Emerson et al., 2019). From these findings and our own preliminary batch experiments (Supplementary Figure S5 in Supplementary Data Sheet S2), we also expected enhanced cell growth in our bioreactor experiment. Our data confirmed that the use of nitrate as sole N-source is enhancing CO_2_ and H_2_-dependent growth of *C. ljungdahlii* by up to 62% (based on OD_600_) in continuous mode (Figure 3 and Table 2). Emerson et al. (2019) observed 42% increased growth rates for bottle experiments with CO_2_ and H_2_, while the pH increased from 6.0 to 8.0. We observed a similar increase in the pH-value and a ∼200% increased OD_600_ in our preliminary bottle experiments (Supplementary Figure S5 in Supplementary Data Sheet S2). All our bioreactors with nitrate feed had high OD_600_-values even at low pH values, whereas the ammonium bioreactors showed a correlation between low pH and low OD_600_ (Tables 1–3). We had not anticipated this uncoupling of pH and OD_600_, because biomass production is becoming limited at lower pH (Richter et al., 2016). Consequently, less acetate is produced from acetyl-CoA and, in turn, less ATP is available for the Wood-Ljungdahl pathway (Schuchmann and Müller, 2014). One possible explanation for this observation is that the depleting pool of ATP at a low pH is refilled with ATP generated through the reduction of nitrate and concomitant redirection of reducing equivalents. This ATP can then be used for biomass formation.

Our data show that the highest OD_600_ in our bioreactors with nitrate feed ranged between an OD_600_ of 1.29 and 1.36 at a medium feed rate of 0.10 mL min^–1^ (Table 2), and an OD_600_ of 1.21 and 1.49 at a medium feed rate of 0.19 mL min^–1^ during different periods (Table 3). This indicates that ATP was not the limiting factor for growth for the bioreactors with nitrate feed. Thus, nitrate reduction, on the one hand, was sufficient to regenerate redox cofactors, and on the other hand, provided more ATP for biomass formation. Ethanol formation was neither observed in our bottle experiments nor in the experiments by Emerson et al. (2019). This led to the hypothesis by Emerson et al. (2019) that *C. ljungdahlii* predominantly shifts electrons into nitrate reduction rather than toward ethanol formation. Noteworthy, however, is that the generated ammonium was responsible for an increasing pH-value. Here, we demonstrated for all bioreactors with nitrate feed that ethanol production was still possible when the pH was controlled to lower values, which rejects the hypothesis by Emerson et al. (2019) (Figures 3B–5B). We theorize here that ethanol formation was absent in the bottle experiments due to the increasing pH-value from ammonium production, which we were able to prevent with the bioreactors (Supplementary Figure S5). Again, this shows that observations with bottles should be followed up with pH-controlled bioreactors.

### Nitrite Accumulation Indicated a Metabolic Crash of *C. ljungdahlii*

All bioreactors with nitrate feed showed different performance behavior during continuous mode (Figures 3–5). We observed crash events for nitrate bioreactors in which we did not force a decrease of the pH by feeding acid, but let the pH decrease by means of microbial acetate production (experiments 1 and 2) (Figure 3, 4). These crashes were stochastic, because they occurred at different time points of the cultivation. This was independent of the bioreactors, because we had already observed the reproducible nature of our MBS in our preliminary experiment with ammonium feed (Figure 2). For each nitrate bioreactor that crashed, we measured an accumulation of nitrite and nitrate at the time point when the crash occurred and afterward (see Supplementary Data Sheet S3). Before the crashes, we were not able to detect nitrate in any sample. Therefore, we assume that the applied nitrate feed rates of 0.11 mmol h^–1^ (18.7 mM × 0.10 mL min^–1^) for the first nitrate experiment (bioreactor 4/5/6) and 0.21 mmol h^–1^ (18.7 mM × 0.19 mL mnvalues, whereas the ammonium bioreactors showed a correlationi^1^) for the second and third nitrate experiment (bioreactor 7/8/9/10) was lower than the metabolic uptake rate for nitrate of *C. ljungdahlii*. However, our results indicate that an accumulation of nitrite and nitrate above a certain threshold is harmful to the microbes and leads to an abrupt halt of the metabolism for yet unknown reasons. A complete physiological characterization of the nitrate metabolism of *C. ljungdahlii*, or any other acetogen, is still missing in literature. Emerson et al. (2019) described that once the applied nitrate was depleted, the culture halted acetate production and crashed (as measured by the OD_600_). The authors explained the crash with an abrupt end of the ATP supply, which is critical to maintain high cell densities for *C. autoethanogenum* (Valgepea et al., 2017). However, the bottle cultures of Emerson et al. (2019) did not crash completely. The OD_600_ decreased by 50% but recovered after a short lag phase, indicating that the remaining CO_2_ and H_2_ was further consumed. An accumulation of nitrite was neither observed during the crash in these experiments nor in our own preliminary bottle experiments (Supplementary Figure S5 in Supplementary Data Sheet S2). One explanation might be that the metabolic crash was triggered by an insufficient regeneration of NADH. *C. ljungdahlii* possesses two putative hydroxylamine reductases (CLJU_c22260 and CLJU_c07730), which could catalyze the reduction of nitrite to ammonium with electrons from NADH (Köpke et al., 2010; Nagarajan et al., 2013). Since we observed simultaneous nitrite and nitrate accumulation in crashing cultures, a metabolic bottleneck at this catalytic step is possible. Another explanation might be that nitrite and/or nitrate inhibit one or several enzymes in *C. ljungdahlii*. Then, as soon as some nitrite and/or nitrate accumulated and inhibited the metabolism, a feedback loop was triggered that quickly led to a complete crash of the metabolism.

For recovering the culture, we assume that the inhibiting compounds have to be washed out of the system to a certain critical threshold. In addition, some removal of the inhibiting compounds due to the recovering activity of the culture would also contribute. For bioreactor 4, we observed a constant decrease of the OD_600_ and acetate and ethanol production rates after the crash in Period I (Figure 3). However, on day 13–14 the decrease started to reach a valley, which indicates that the microbial growth was able to catch up with the dilution of our continuous process. For this bioreactor, the pH in Period II was still high enough to support sufficient growth, and after ∼2 HRT periods the growth rate of the microbes exceeded the dilution rate and the OD_600_ increased again (Figure 3A). The recovery of this bioreactor 4 in growth as well as in acetate and ethanol production rates indicates that: (1) the nitrate reduction pathway is not *per se* inhibited at low pH; and (2) the reduction of nitrate and the production of ethanol is possible simultaneously, and that most likely the low pH triggers a thermodynamic shift toward ethanol production (Richter et al., 2016). However, it remains elusive why the ethanol/acetate ratio in bioreactor 4 reached a nearly 10-fold higher value after recovering from the crash in the presence of nitrate compared to the bioreactors with ammonium feed (Figure 3B and Table 2). Importantly, applying a higher feed rate and similar cultivation conditions with another set of bioreactors for experiment 2 reached only a maximum ethanol/acetate ratio of 1.4 (Table 3). In contrast, the crash occurred for bioreactor 5 in Period II. While the OD_600_ immediately decreased after the crash, the acetate and ethanol production rates remained somewhat constant until the switch to Period III. However, this bioreactor 5 never recovered from the crash in terms of OD_600_. We believe that the lower pH levels during Period III for bioreactor 5 prevented the growth recovery, which for bioreactor 4 took place at the higher pH level of Period II. We had found reduced growth conditions for bioreactors with ammonium at the lower pH levels, indicating that the growth rate is constantly decreasing while decreasing the medium pH (Figure 2, Supplementary Figure S4 in Supplementary Data Sheet S2, and Table 2). The same findings hold true for bioreactor 6 for which the crash occurred even later in the cultivation. When we applied the 90% higher medium feed rate but kept the same pH maintenance conditions for bioreactor 7 and bioreactor 8 for experiment 2, single crash events still occurred, but at later time points (Figure 4).

What could be the reason for the stochastic crashes? Valgepea et al. (2017) discussed occurring “crash and recover cycles” during syngas fermentation with *C. autoethanogenum*. They hypothesized that the Wood-Ljungdahl pathway becomes the limiting factor during a period of ample supply of acetyl-CoA at higher biomass and acetate concentration. This can result in an insufficient supply of reducing equivalents due to a loss of H_2_ uptake when the Wood-Ljungdahl pathway cannot keep up anymore. Consequently, the cells are not able to deliver the ATP demand, resulting in a crash. The cells recovered once the extracellular acetate concentration went below a certain threshold but crashed again after exceeding the threshold. Unfortunately, these threshold acetate concentrations were not given.

We observed higher acetate production rates for bioreactor 4 and 6 before the crash, compared to those of the bioreactors with ammonium feed (Figures 2B, 3B). Bioreactor 5 did not reach a similarly high acetate concentration, but the crash occurred at the beginning of Period IV at the lower pH of 4.5. Intrinsically, the extracellular acetate concentration would be higher as a key to trigger the crash event. We assume a similar correlation of high acetate concentration and low pH for bioreactor 7 and bioreactor 8, which had a similar acetate production rate compared to bioreactor 5 at the time point of the crash event (Figure 4).

### A Sensitive pH-Environment Based on an Interplay Between Undissociated Acetic Acid and Ammonium Increased Growth and Ethanol Production Rates

To further tackle the question of why we observed the crash events, we applied active pH maintenance with base and acid feed in our third experiment (bioreactor 9 and 10) (Figure 5). This immediate adjustment of the pH environment influenced growth, and acetate and ethanol production rates. At a pH of 5.5 (Period II), it caused stagnation of growth and acetate production rates, while simultaneously ethanol production started to increase. In contrast, the slow decrease of the pH in Period II due to microbial acetic acid production in bioreactor 7 and 8 accelerated ethanol production by 2–4-fold and biomass production by 11–28% compared to bioreactor 9 and 10, while acetate production first increased, but then quickly decreased (Figure 4). We believe that nitrate-reducing cells of *C. ljungdahlii* generate a sensitive pH-environment based on the buffering effect of the interplay between undissociated acetic acid production and ammonium production. This enables a more efficient pH balance and electron flow toward biomass production, and more reduced fermentation products, such as ethanol, but at the cost of a highly unstable environment. Small perturbations to the system seem to lead to a severe disbalance and immediate crash of the microbial growth. By feeding acid to actively lower the pH, this highly unstable environment can be controlled better, but at the cost of lower biomass and ethanol production rates. Without actively decreasing the pH with acid feed, the pH-environment remains sensitive to external influences. This could also explain the partly increasing pH-values during periods of higher (unbalanced) ammonium production with respect to acetate production. By a detailed look into literature and to the best of our knowledge there is no study that describes a similar pH effect for an acetogen. Our experiments showed highest ethanol production rates for nitrate-reducing cultures of *C. ljungdahlii* at pH 5.6 under fully controlled pH conditions. This indicates that optimum production conditions exist, and it will be of particular interest to maintain the bioreactors at this pH for longer cultivation times in future experiments (Tables 2,  3).

In conclusion, nitrate reduction offers a great potential to further optimize gas fermentation of *C. ljungdahlii*. Because ATP limitation is one of the highest burdens to overcome for acetogens (Schuchmann and Müller, 2014; Molitor et al., 2017), the surplus of ATP derived from nitrate reduction could be used to extent the product portfolio toward energy-intense products (Emerson et al., 2019). However, our work clearly demonstrates that nitrate metabolism of *C. ljungdahlii* needs further investigation on both a physiological and a bioprocessing level. The stochastic metabolic crashes demonstrate the importance of replicated bioreactor experiments in the field of acetogen research.

## Data Availability Statement

The datasets generated for this study are available in the Supplementary Tables S2–S6 in Supplementary Data Sheet S3.

## Author Contributions

C-MK and LA designed the MBS. LA, C-MK, and BM planned the experiments. C-MK and NK-K built, maintained, and sampled the bioreactors. LA and BM supervised the project. C-MK analyzed the raw data and drafted the manuscript. All authors edited the manuscript and approved the final version.

## Conflict of Interest

The authors declare that the research was conducted in the absence of any commercial or financial relationships that could be construed as a potential conflict of interest.
