# Supplementary material for: Nitrate Feed Improves Growth and Ethanol Production of Clostridium ljungdahlii With CO2 and H2, but Results in Stochastic Inhibition Events
Source: Front Microbiol. 2020 May 6;11:724. doi: 10.3389/fmicb.2020.00724 (PMC7219301; doi:10.3389/fmicb.2020.00724)

Project overview

Project name: MBS frame blueprint

Project number: 40afc9d6b7ecec6553ffad72df53b7871

i24.ws?40afc9d6b7ecec6553ffad72df53b7871

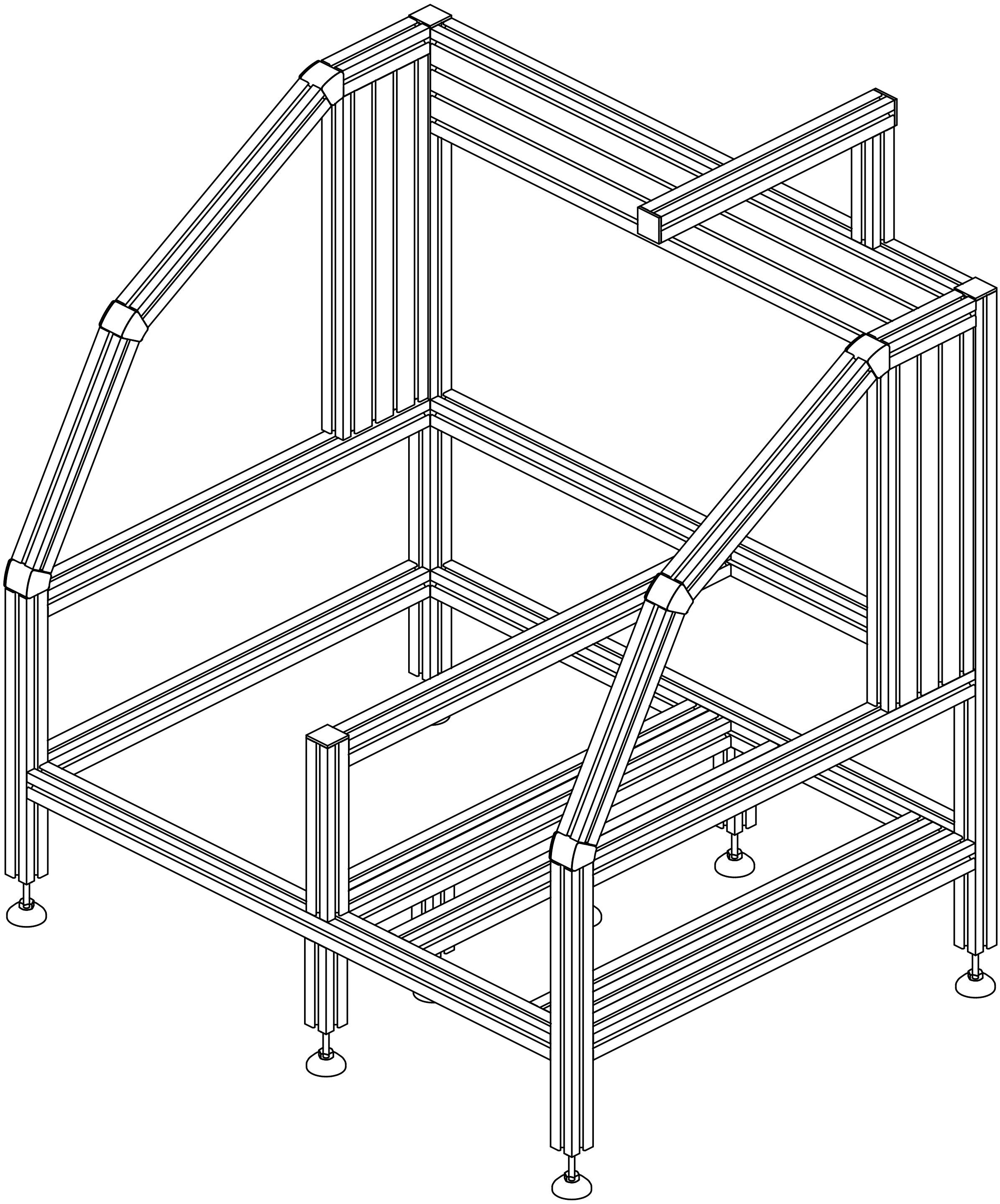

Assembly overview

Project overview 1
Assembly overview 2
Parts list 3
Profile machining processes 4

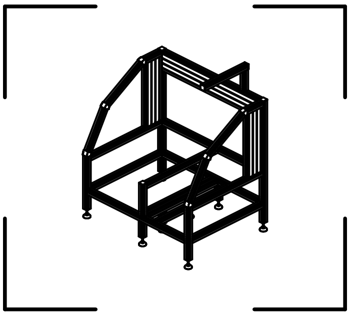

Assembly 1

Isometric view 12
Multiview projection 13
Exploded view 15
Installation guide 16

+2 Separately placed articles

Parts list

Position	Article designation	Article No.	Side	Quantity (All together)	Quantity (Assembly 1)	Quantity (Separately)
1	Profile 6 120x30, natural, Length: 396.51mm	0.0.419.04	-	2	2	-
2	Profile 6 120x30, natural, Length: 740mm	0.0.419.04	-	1	1	-
3v	Profile 6 30x30, natural, Length: 100mm	0.0.419.01	6	4	4	-
4	Profile 6 30x30, natural, Length: 123.7mm	0.0.419.01	-	2	2	-
5	Profile 6 30x30, natural, Length: 130mm	0.0.419.01	-	1	1	-
6	Profile 6 30x30, natural, Length: 201mm	0.0.419.01	-	1	1	-
7	Profile 6 30x30, natural, Length: 250mm	0.0.419.01	-	2	2	-
8	Profile 6 30x30, natural, Length: 300mm	0.0.419.01	-	2	2	-
9	Profile 6 30x30, natural, Length: 330mm	0.0.419.01	-	1	1	-
10v	Profile 6 30x30, natural, Length: 330mm	0.0.419.01	8	2	2	-
11	Profile 6 30x30, natural, Length: 540mm	0.0.419.01	-	10	8	2
12	Profile 6 30x30, natural, Length: 740mm	0.0.419.01	-	3	3	-
13v	Profile 6 30x30, natural, Length: 755mm	0.0.419.01	10	2	2	-
14	Profile 6 R30/60-30°, natural, Length: 30mm	0.0.459.54	-	6	6	-
15	Cap 6 30x30, black	0.0.419.22	-	5	5	-
16	Cap 6 R30/60-30°, black	0.0.459.39	-	12	12	-
17	Knuckle Foot D40, M8x80, black	0.0.265.69	-	8	8	-
18	Automatic-Fastening Set 6, bright zinc-plated	0.0.419.71	-	82	82	-

Fastener Technology (All together)

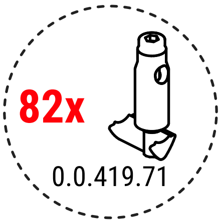

Unmachined profiles

2x Part 1, Profile 6 120x30, natural
Article No.: 0.0.419.04
Length: 396.51mm, Unmachined

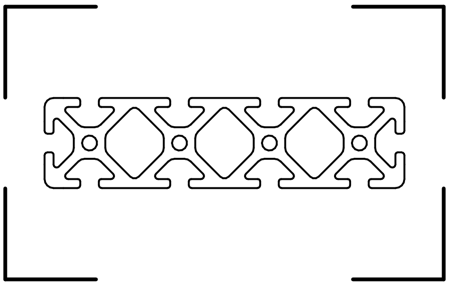

i24.ws?40afc9d6b7ecec6553ffad72df53b7871.1

Part 2, Profile 6 120x30, natural
Article No.: 0.0.419.04
Length: 740mm, Unmachined

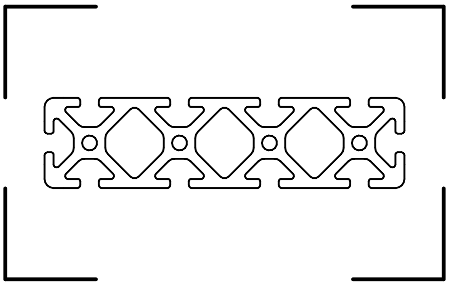

i24.ws?40afc9d6b7ecec6553ffad72df53b7871.2

2x Part 4, Profile 6 30x30, natural
Article No.: 0.0.419.01
Length: 123.696mm, Unmachined

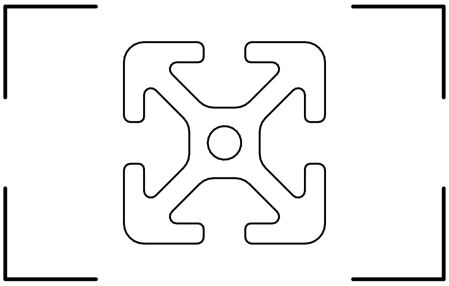

i24.ws?40afc9d6b7ecec6553ffad72df53b7871.4

Part 5, Profile 6 30x30, natural
Article No.: 0.0.419.01
Length: 130mm, Unmachined

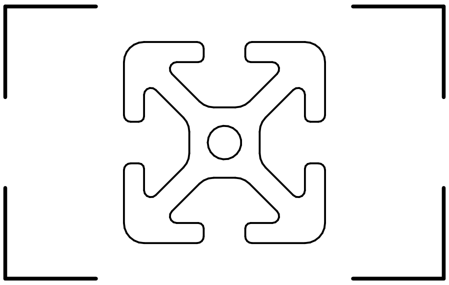

i24.ws?40afc9d6b7ecec6553ffad72df53b7871.5

Part 6, Profile 6 30x30, natural
Article No.: 0.0.419.01
Length: 201mm, Unmachined

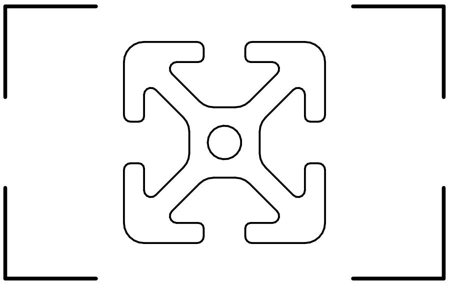

i24.ws?40afc9d6b7ecec6553ffad72df53b7871.6

2x Part 7, Profile 6 30x30, natural
Article No.: 0.0.419.01
Length: 250mm, Unmachined

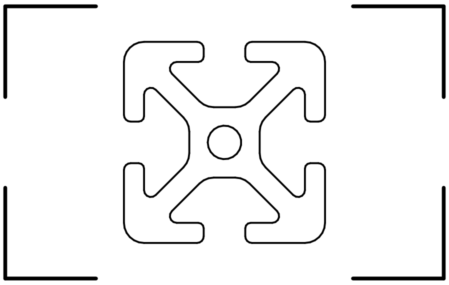

i24.ws?40afc9d6b7ecec6553ffad72df53b7871.7

Unmachined profiles

2x

Part 8, Profile 6 30x30, natural
Article No.: 0.0.419.01
Length: 300mm, Unmachined

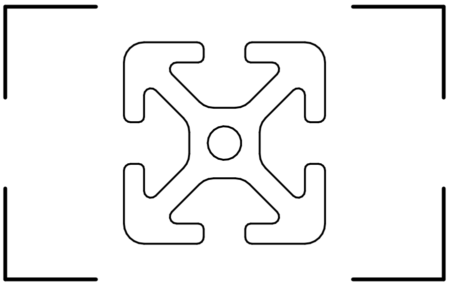

i24.ws?40afc9d6b7ecec6553ffad72df53b7871.8

Part 9, Profile 6 30x30, natural
Article No.: 0.0.419.01
Length: 330mm, Unmachined

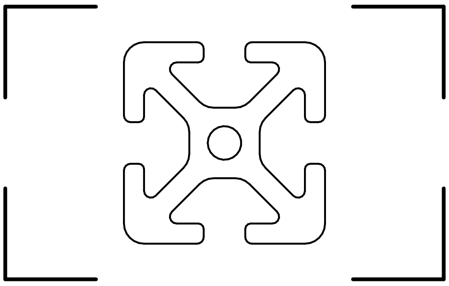

i24.ws?40afc9d6b7ecec6553ffad72df53b7871.9

10x

Part 11, Profile 6 30x30, natural
Article No.: 0.0.419.01
Length: 540mm, Unmachined

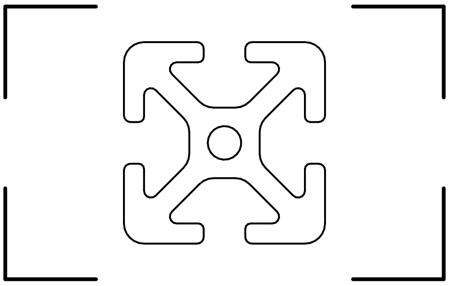

i24.ws?40afc9d6b7ecec6553ffad72df53b7871.11

3x

Part 12, Profile 6 30x30, natural
Article No.: 0.0.419.01
Length: 740mm, Unmachined

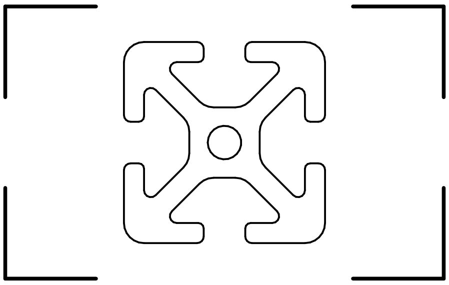

i24.ws?40afc9d6b7ecec6553ffad72df53b7871.12

6x

Part 14, Profile 6 R30/60-30°, natural
Article No.: 0.0.459.54
Length: 30mm, Unmachined

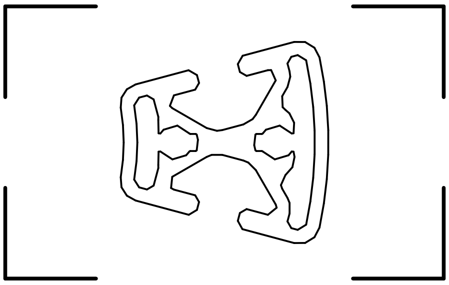

i24.ws?40afc9d6b7ecec6553ffad72df53b7871.14

4x

Machining processes Part 3v, Profile 6 30x30, natural
Article No.: 0.0.419.01, Length: 100mm

View of end face 1

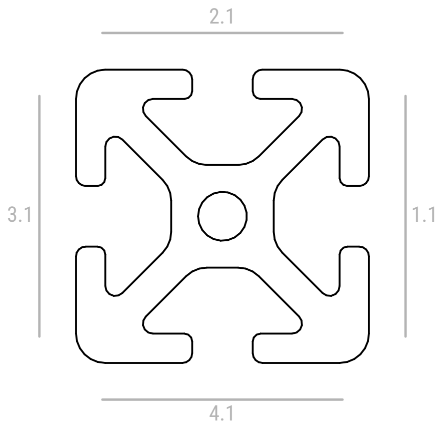

i24.ws?40afc9d6b7ecec6553ffad72df53b7871.3v

Machining type	Side	Number	Dimension (end face 1)	Machining process designation	Dimension (end face 2)
D6.8 drilled hole with M8 thread	End face 1	v1	-	M8x64	-

End face 1

Side 2
is at the top

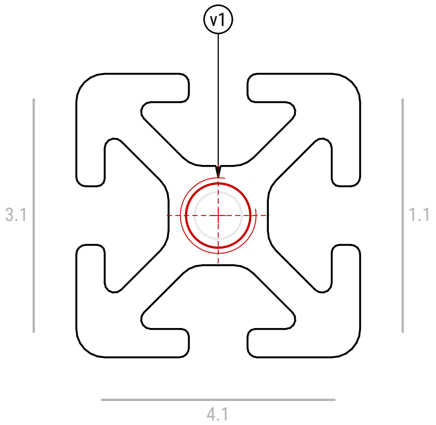

M8x64

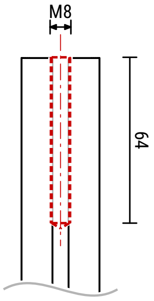

v1

Control view (machining processes), part 3v

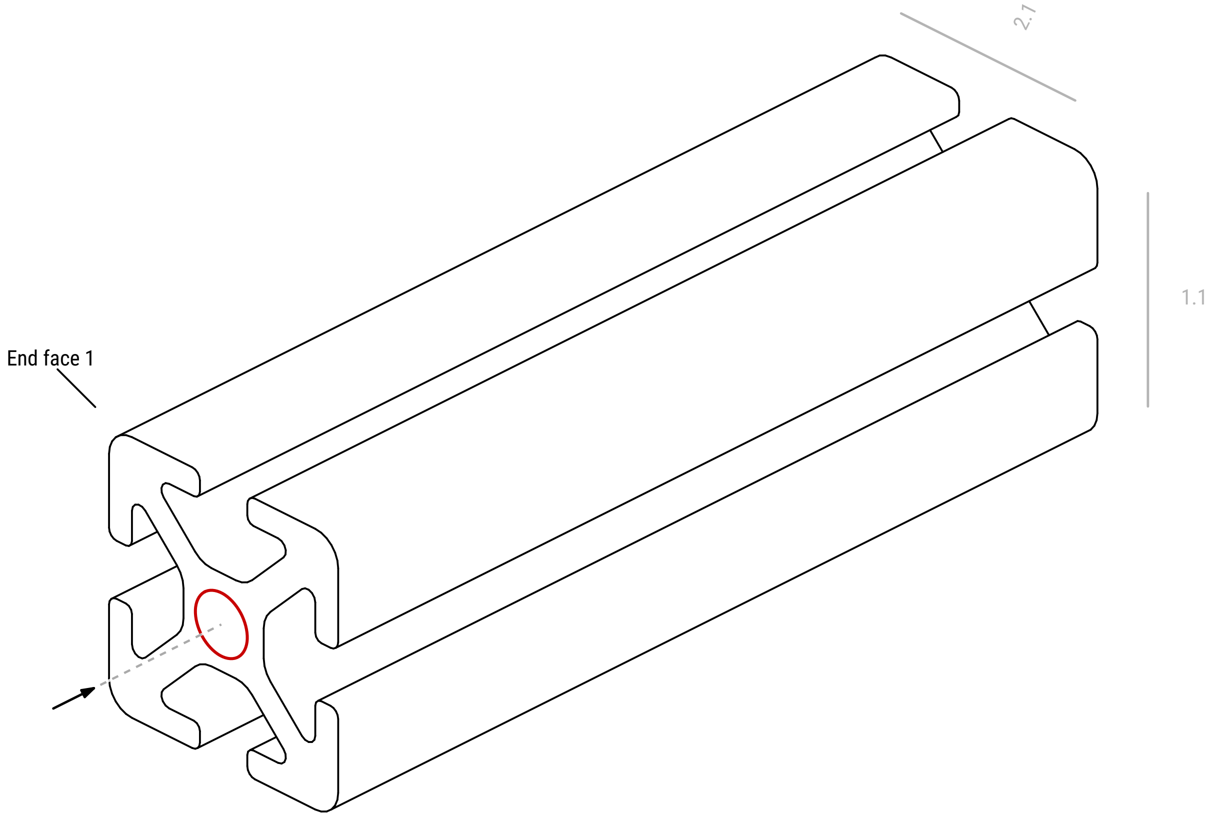

2x

Machining processes Part 10v, Profile 6 30x30, natural
Article No.: 0.0.419.01, Length: 330mm

View of end face 1

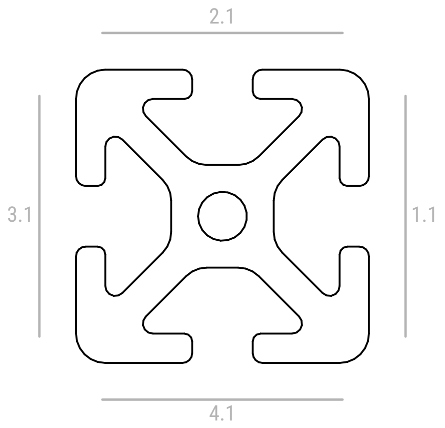

i24.ws?40afc9d6b7ecec6553ffad72df53b7871.10v

Machining type	Side	Number	Dimension (end face 1)	Machining process designation	Dimension (end face 2)
D6.8 drilled hole with M8 thread	End face 2	v1	-	M8x64	-

End face 2

Side 2
is at the top

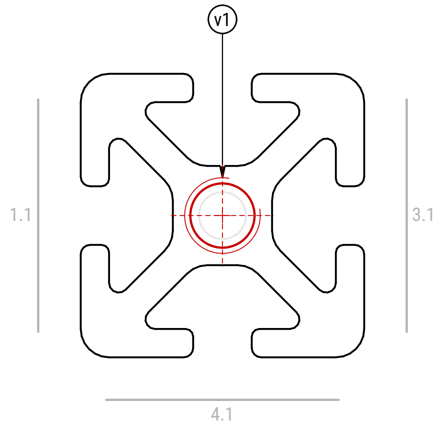

M8x64

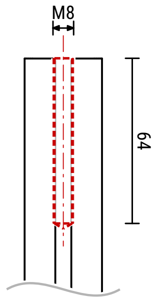

v1

Control view (machining processes), part 10v

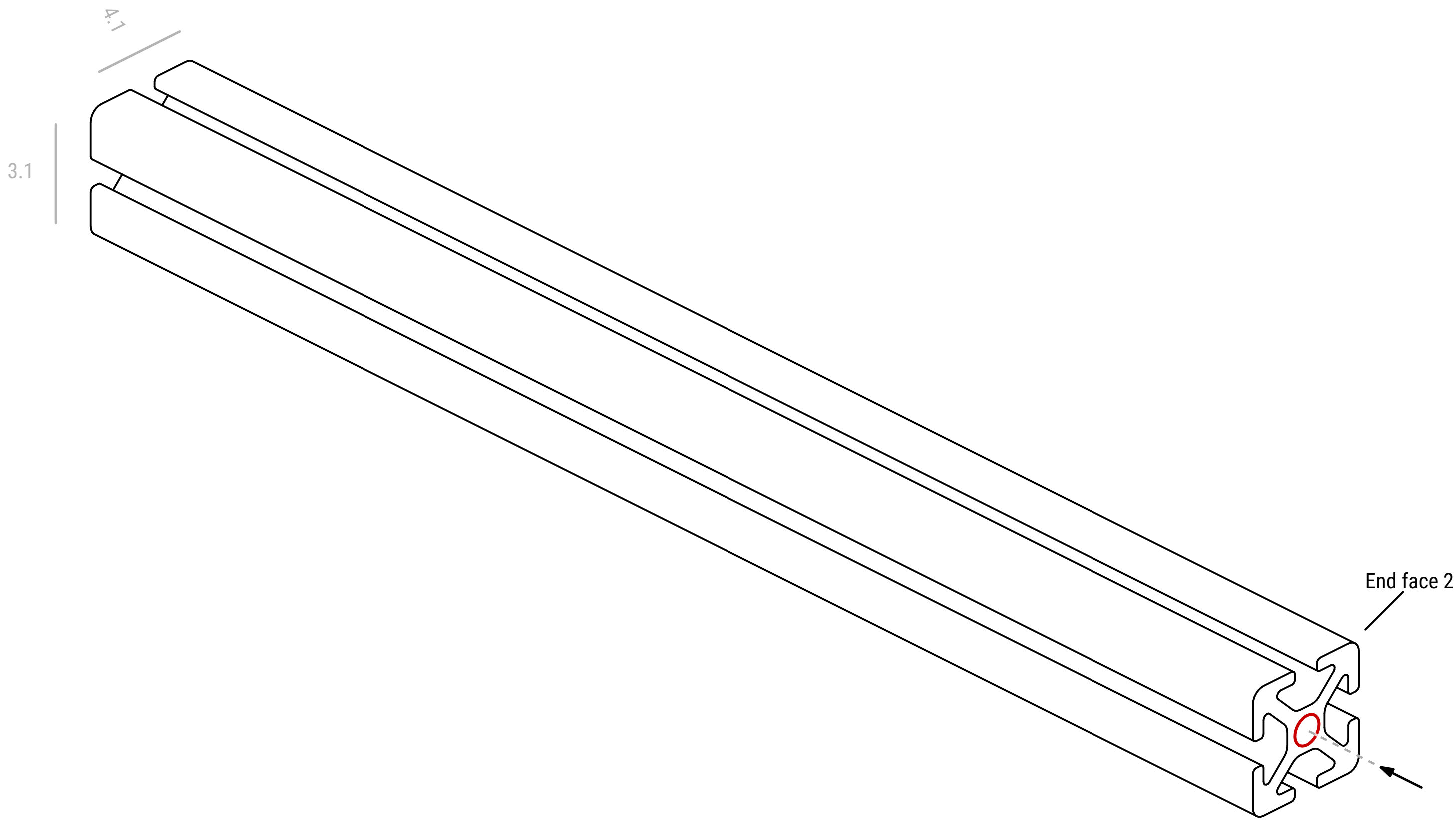

2x

Machining processes Part 13v, Profile 6 30x30, natural
Article No.: 0.0.419.01, Length: 755mm

View of end face 1

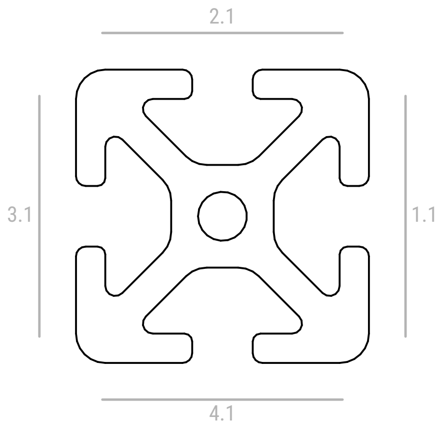

i24.ws?40afc9d6b7ecec6553ffad72df53b7871.13v

Machining type	Side	Number	Dimension (end face 1)	Machining process designation	Dimension (end face 2)
D6.8 drilled hole with M8 thread	End face 2	v1	-	M8x64	-

End face 2

Side 2
is at the top

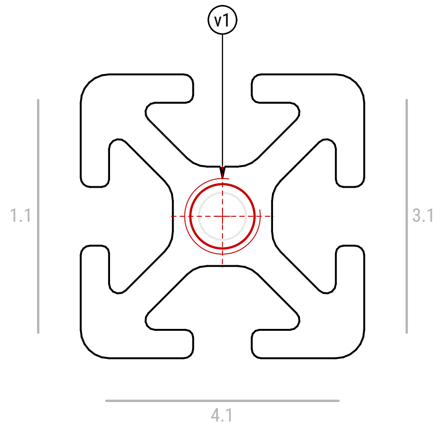

M8x64

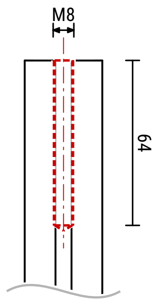

v1

Control view (machining processes), part 13v

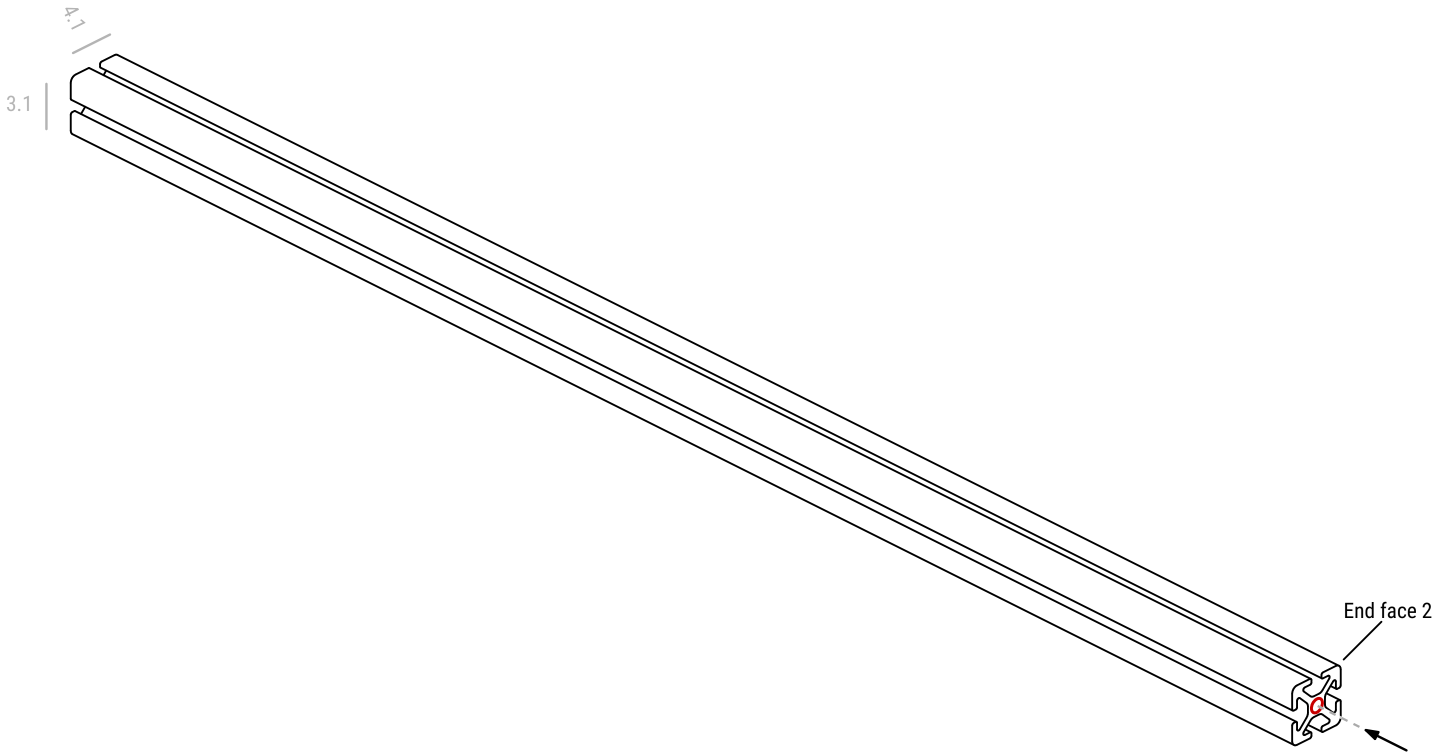

Isometric view

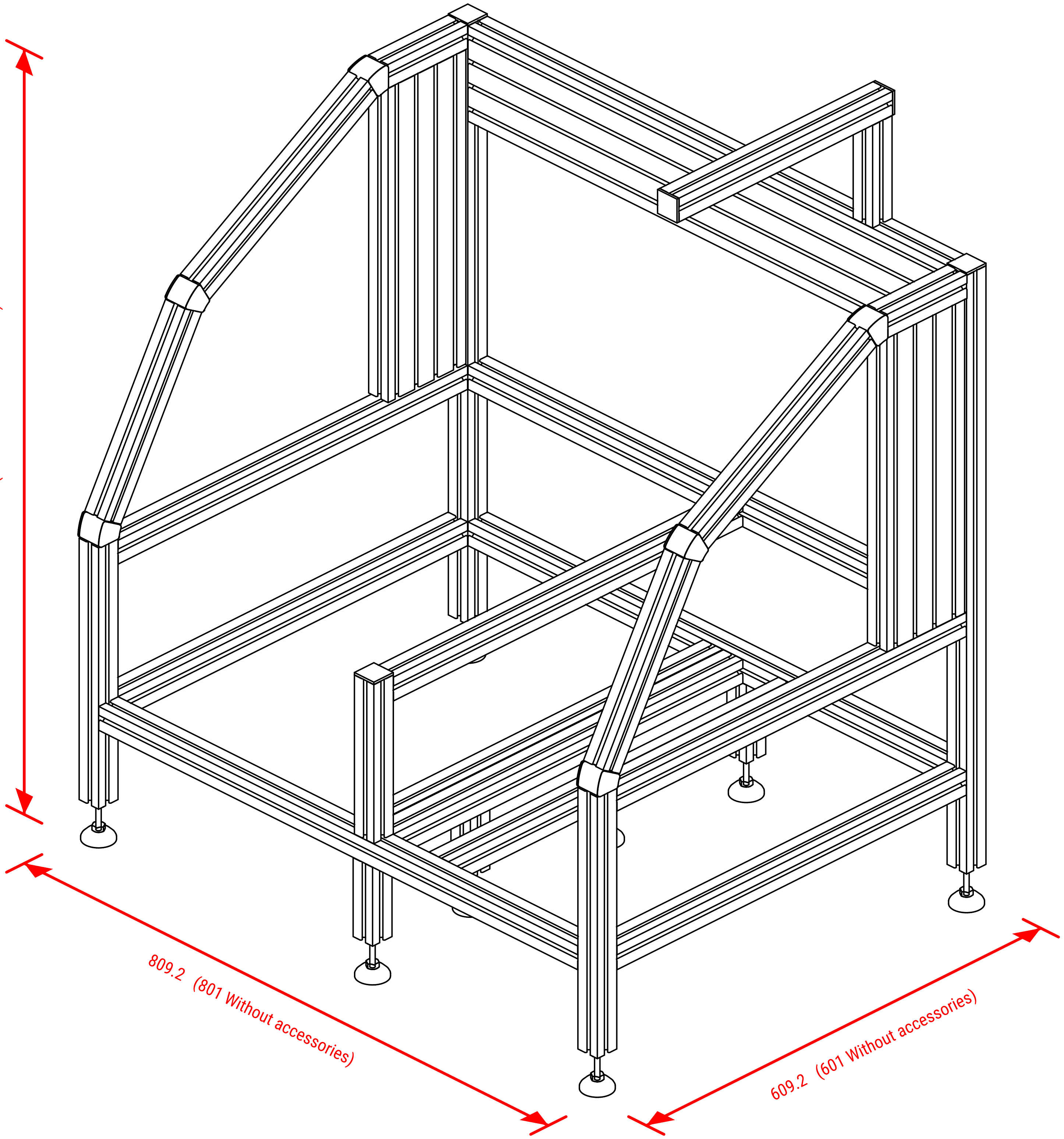

Multiview projection

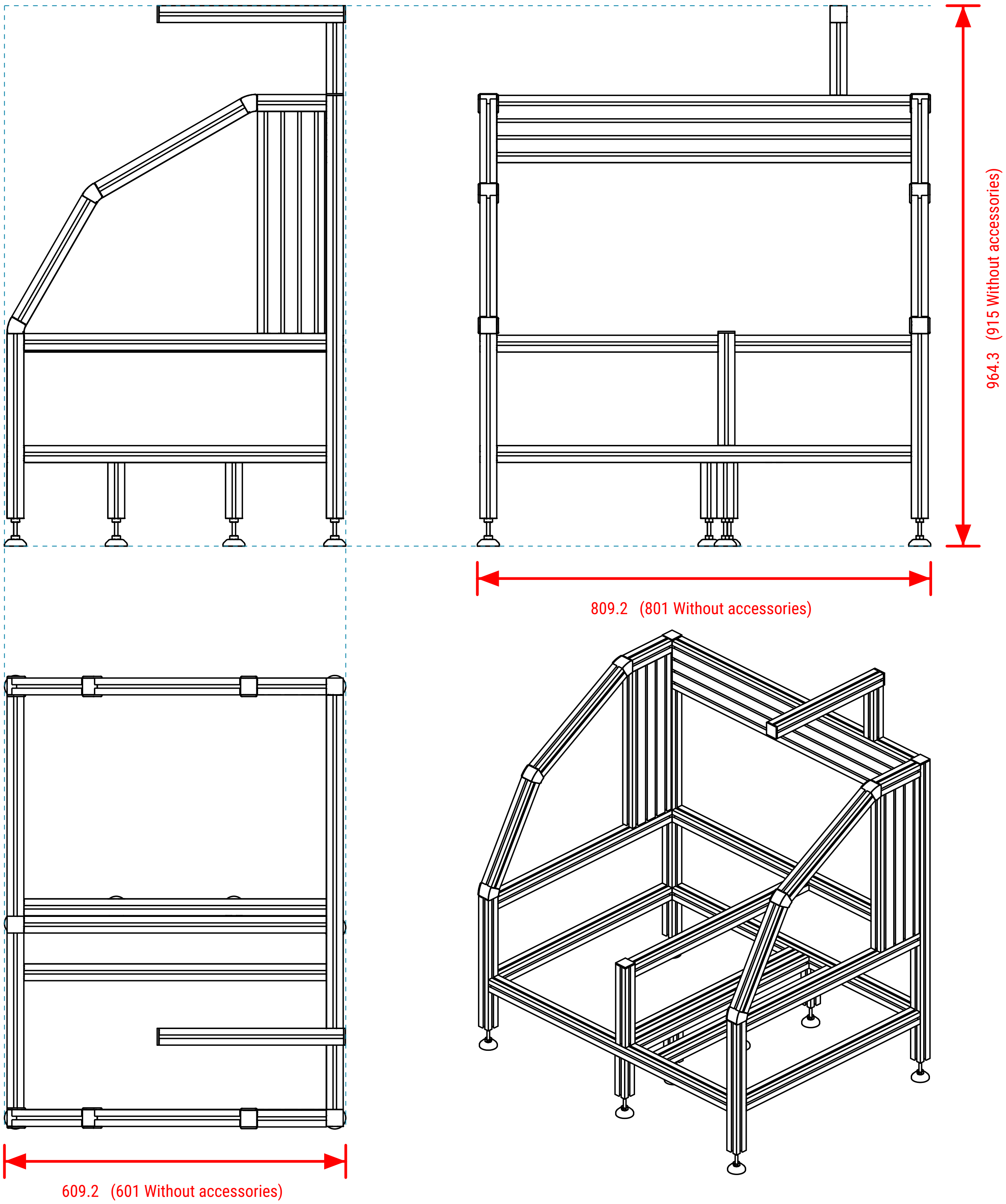

Exploded view (Profiles)

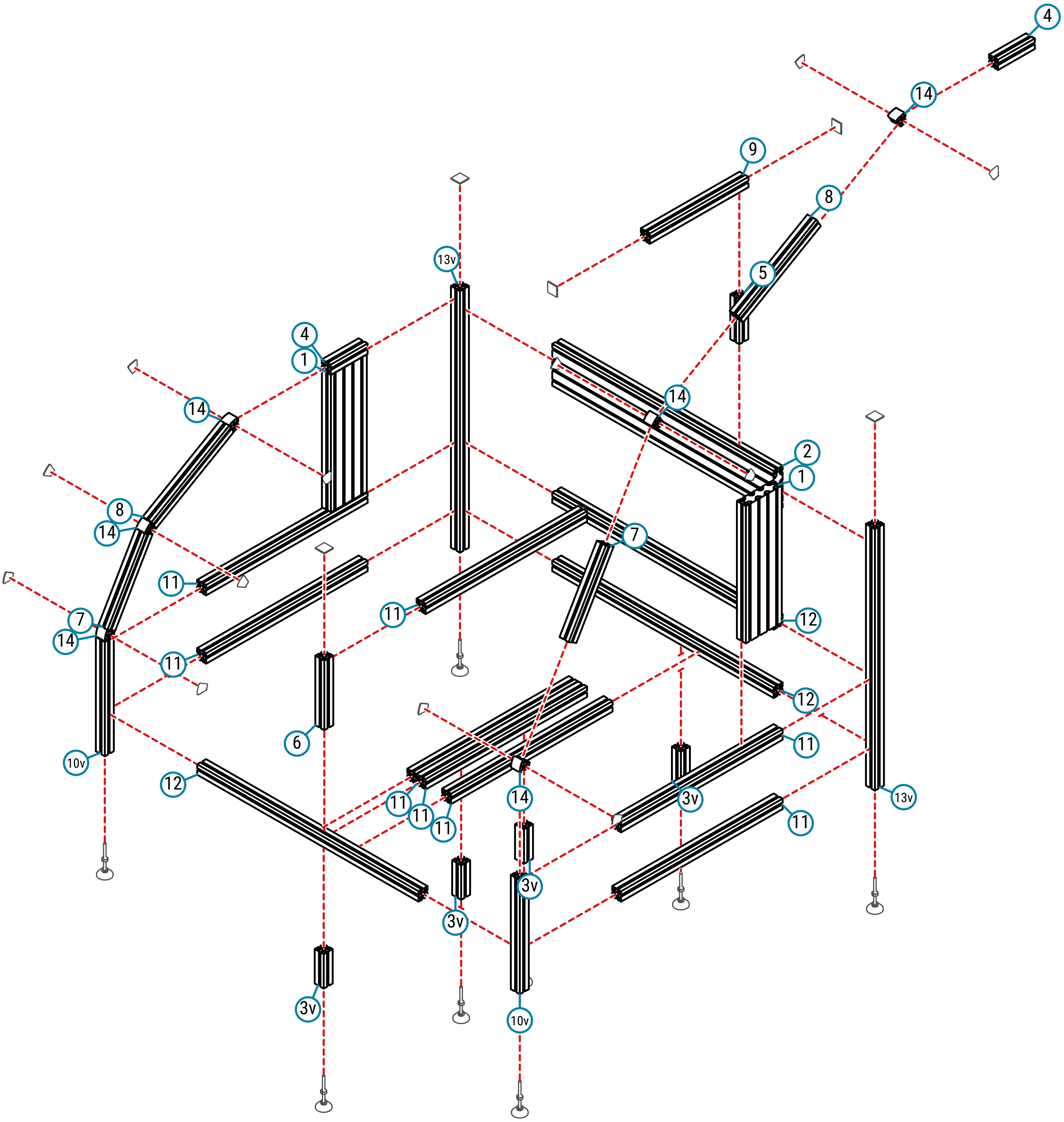

Exploded view (accessories and Fastener Technology)

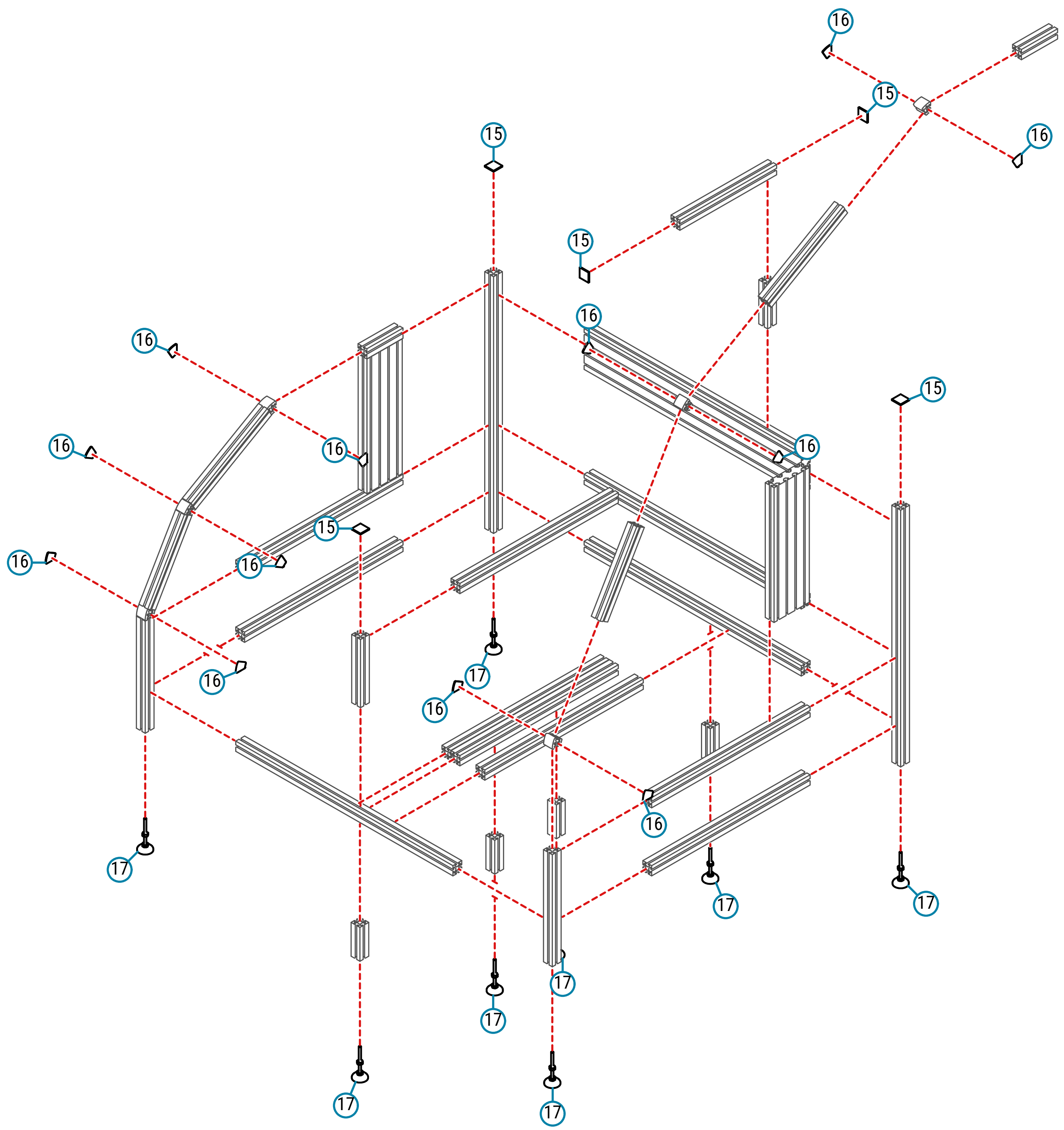

Installation guide

Step 1/ 17

Start with part 16 and 13v

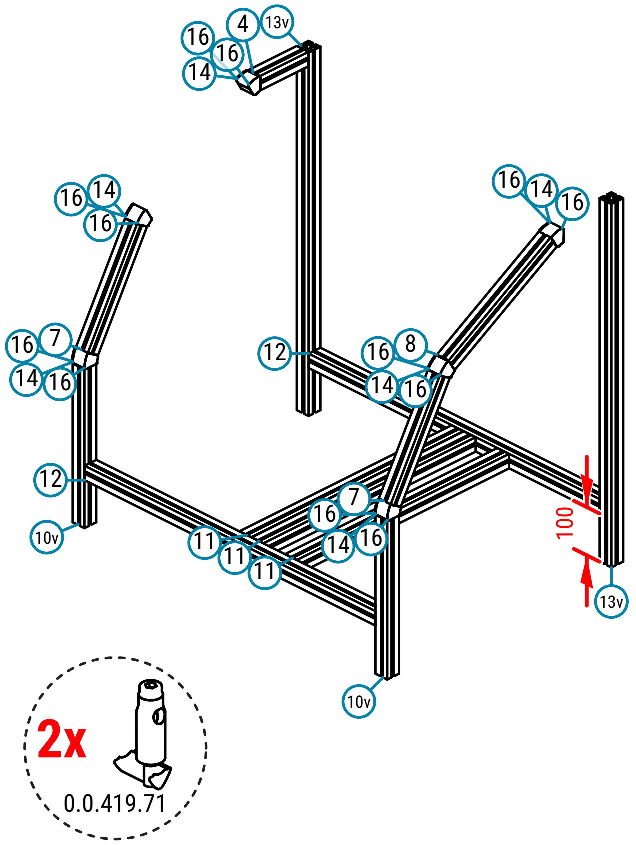

Step 2/ 17

Install 1 x part 11

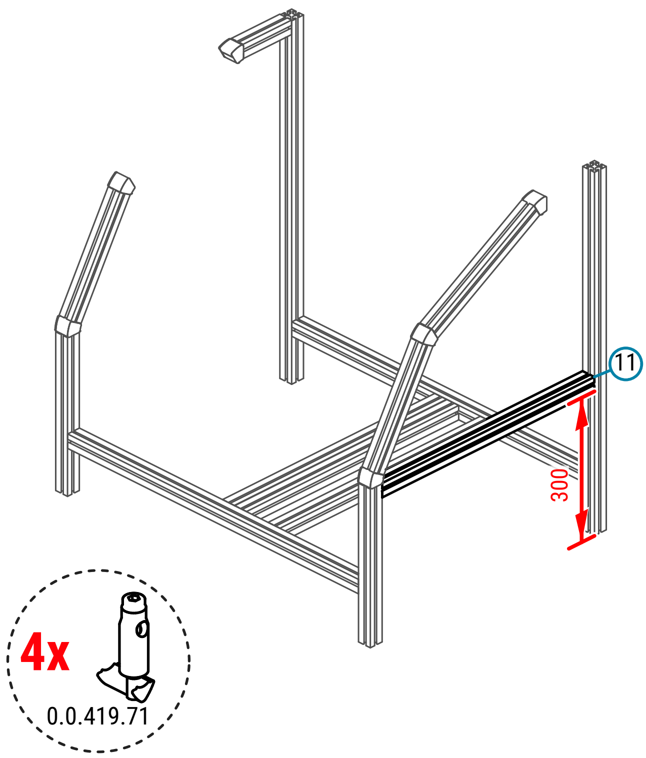

Step 3/ 17

Install 1 x part 8

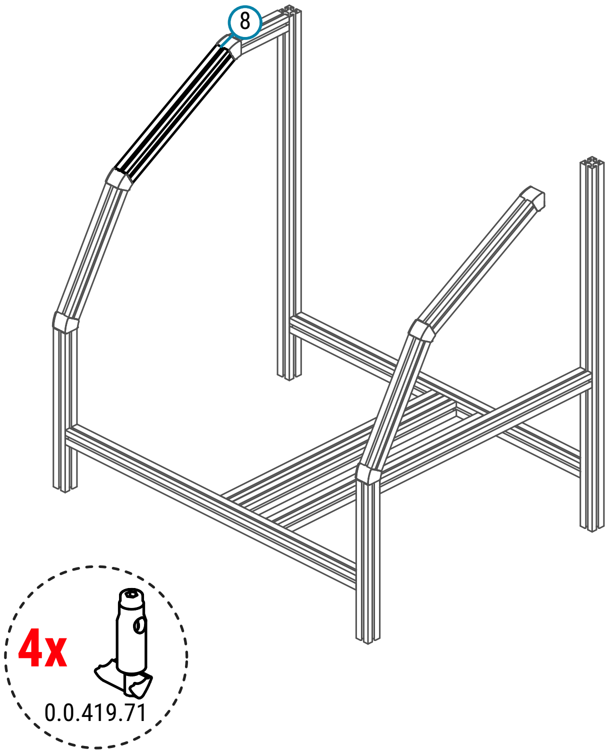

Step 4/ 17

Install 1 x part 12

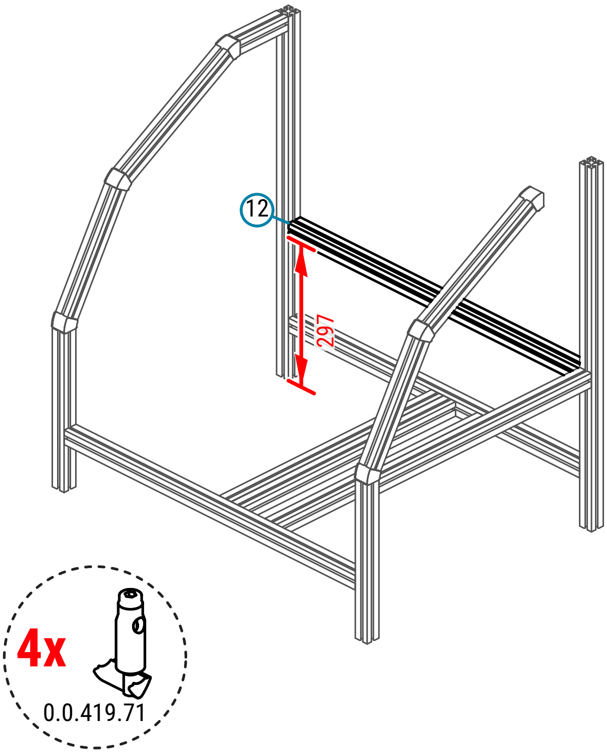

Step 5/ 17

Install 1 x part 2

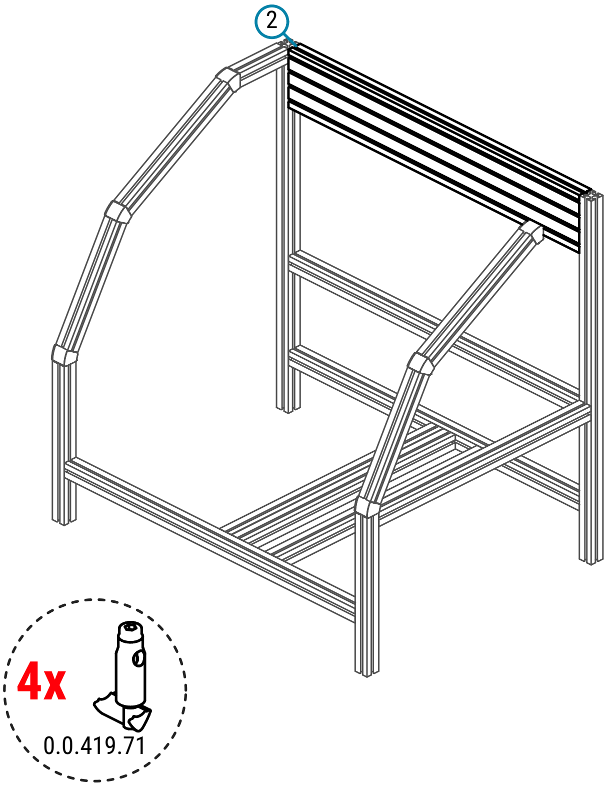

Step 6/ 17

Install 1 x part 1

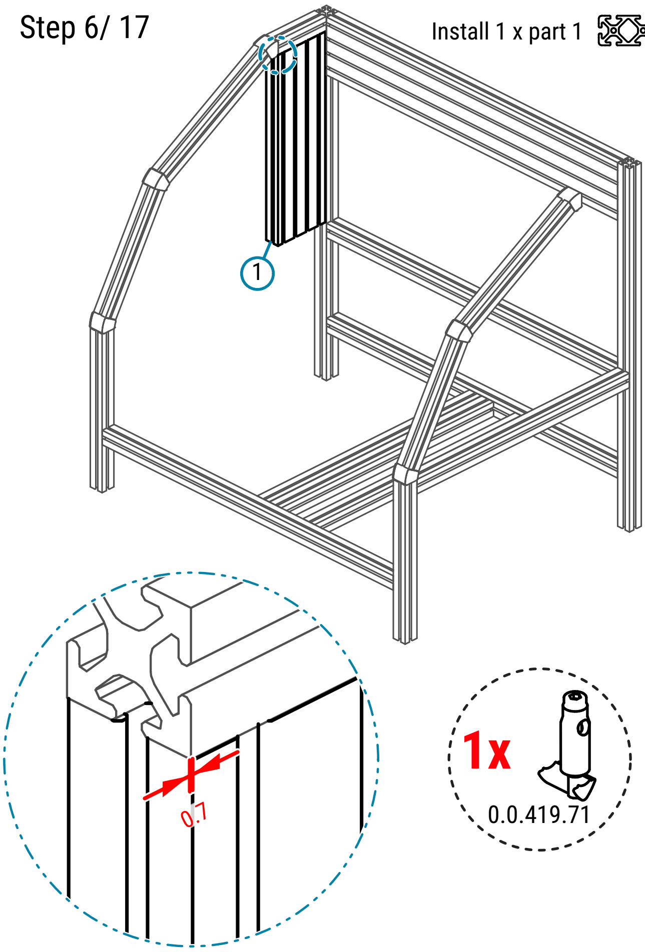

Step 7/ 17

Install 2 x part 11

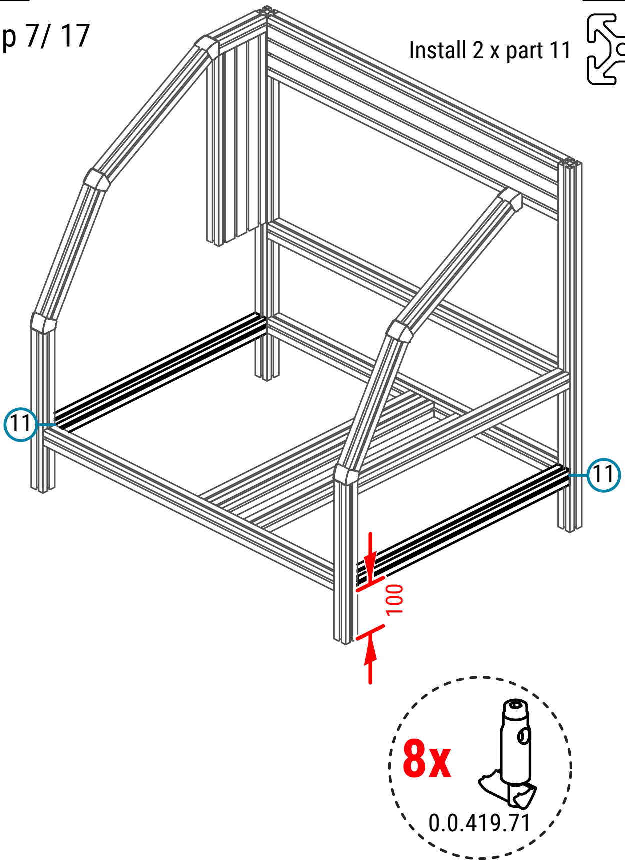

All installed parts have exactly the same dimension

Step 8/ 17

Install 1 x part 11

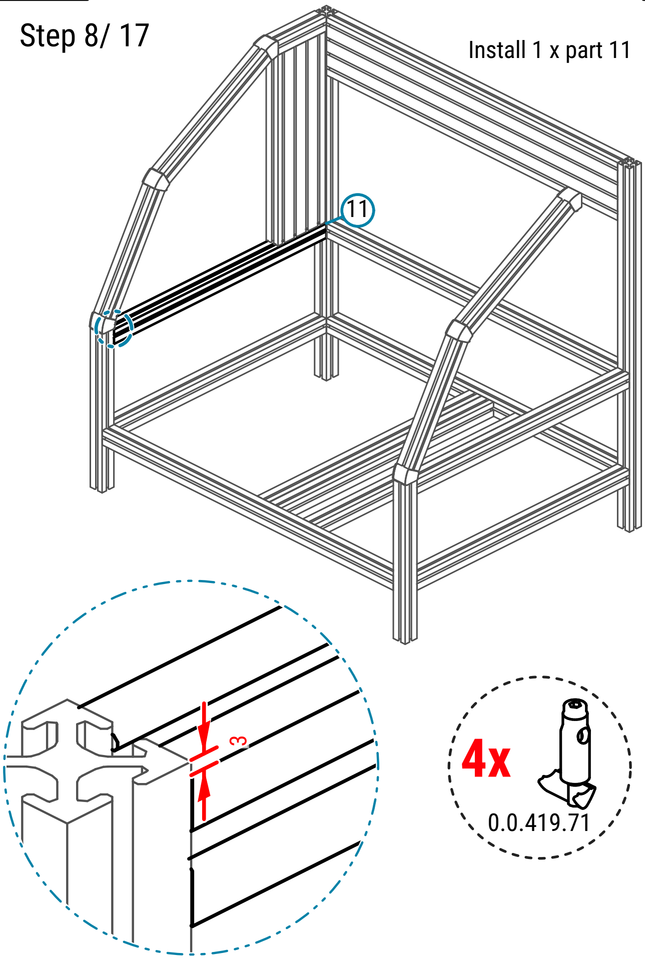

Step 9/ 17

Install 1 x part 1

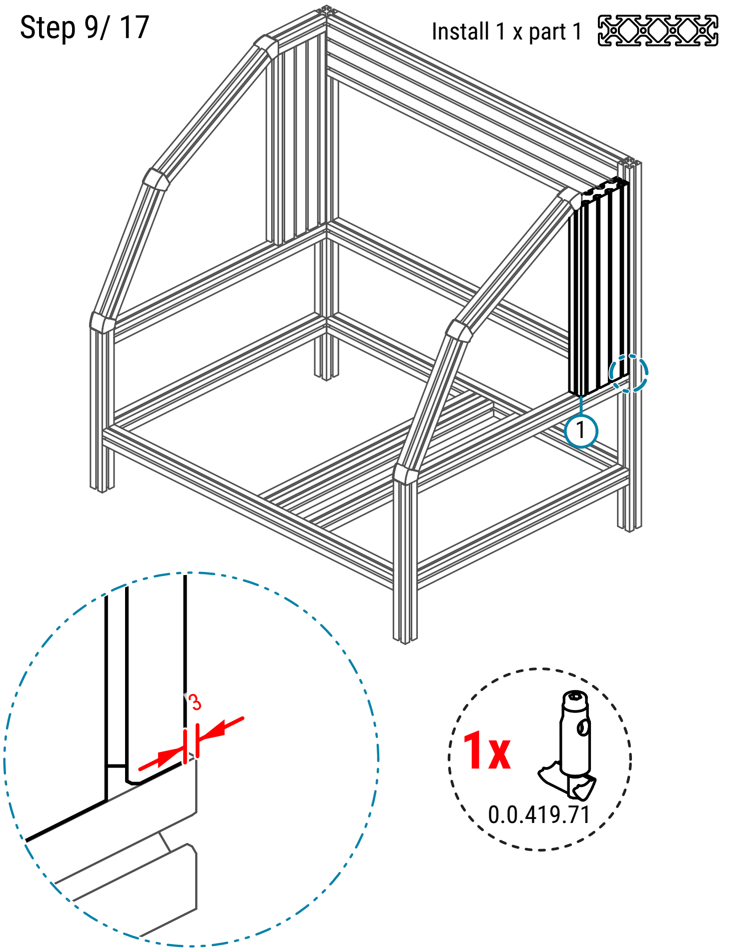

Step 10/ 17

Install 1 x part 6

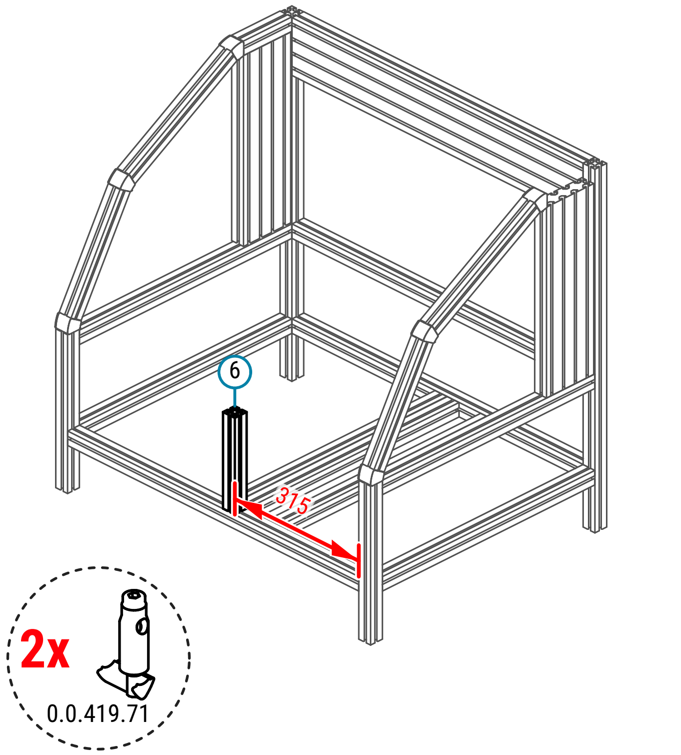

Step 11/ 17

Install 4 x part 3v

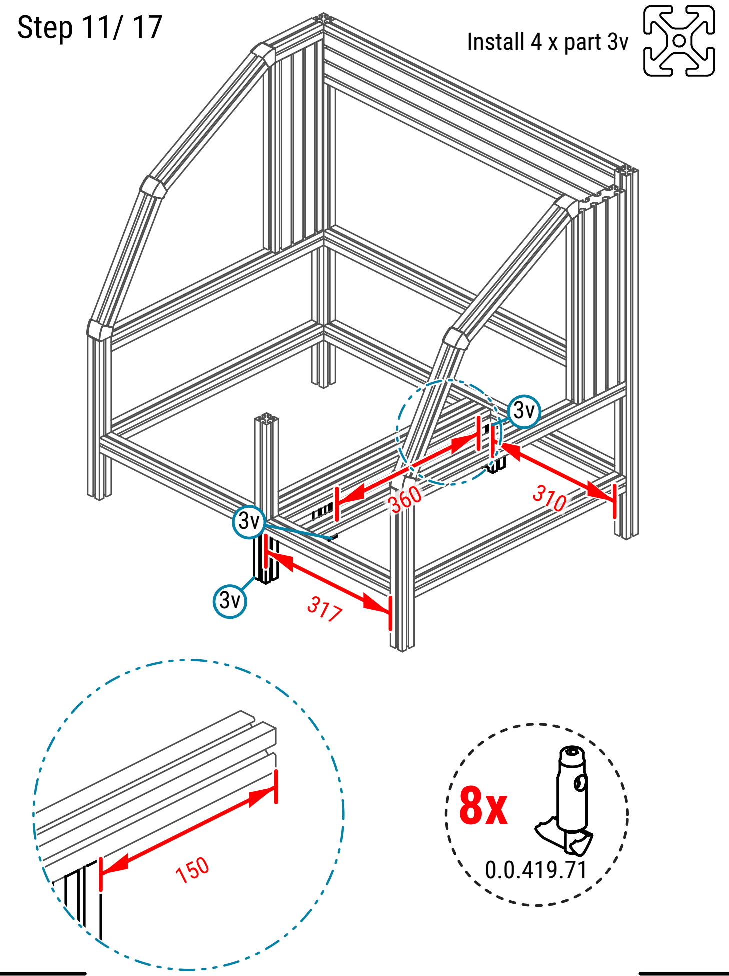

Step 12/ 17

Install 1 x part 5

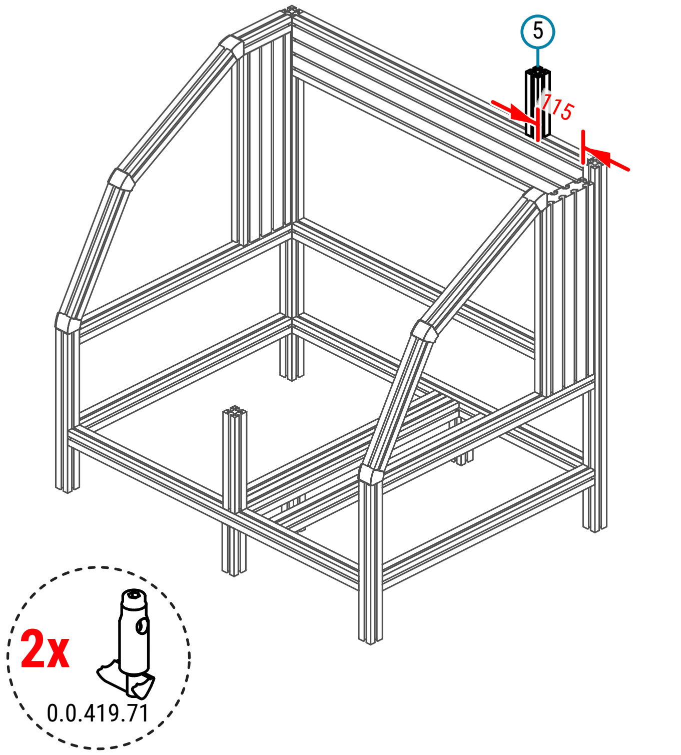

Step 13/ 17

Install 1 x part 9

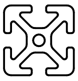

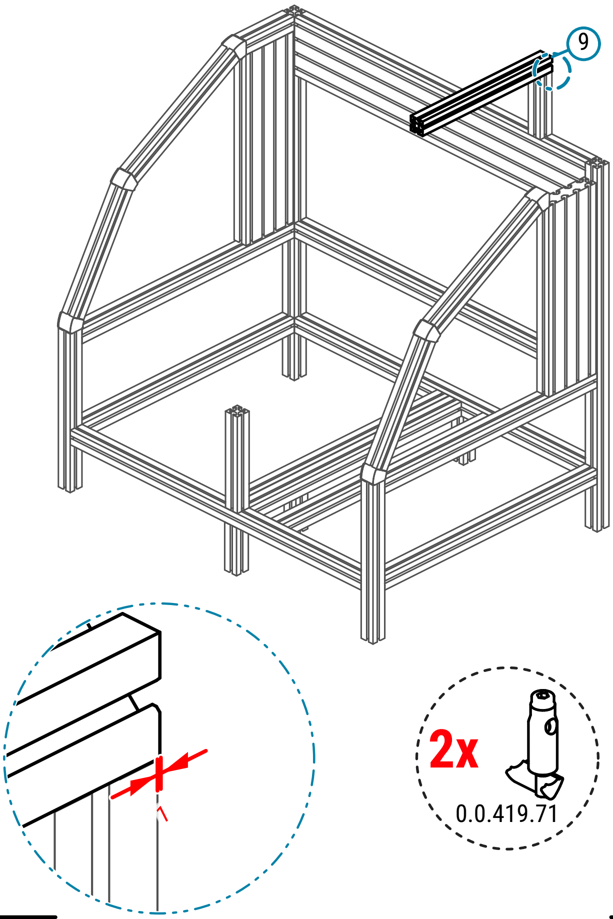

Step 14/ 17

Install 1 x part 4

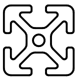

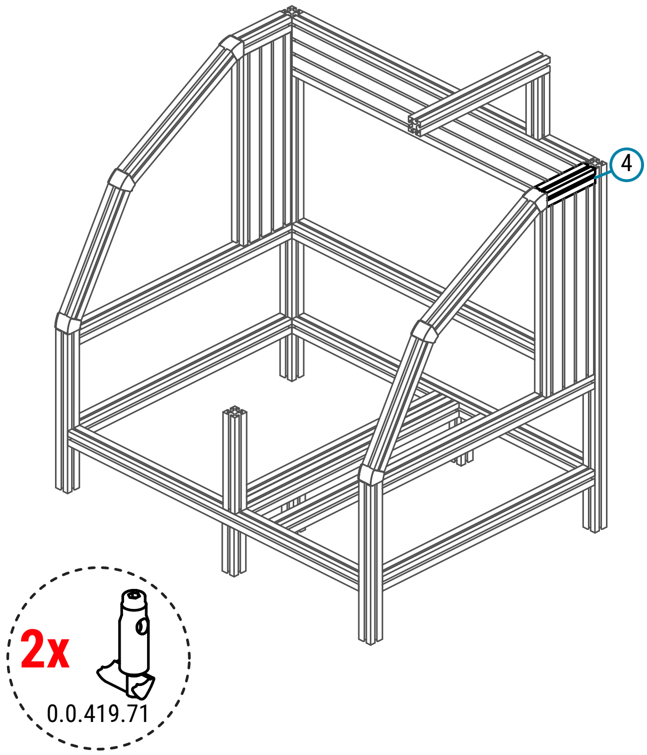

Step 15/ 17

Install 1 x part 11

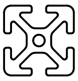

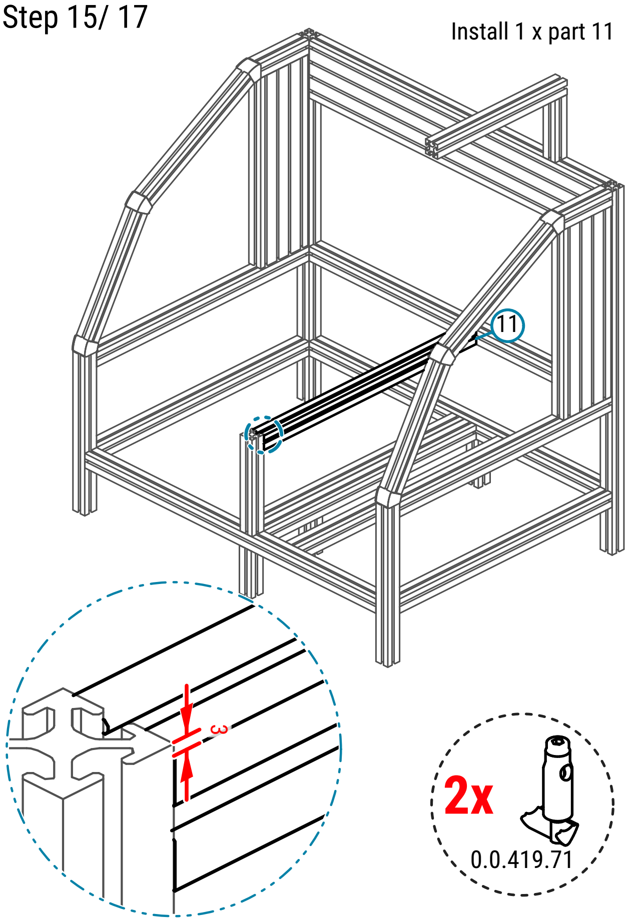

Step 16/ 17

Install 8 x part 17

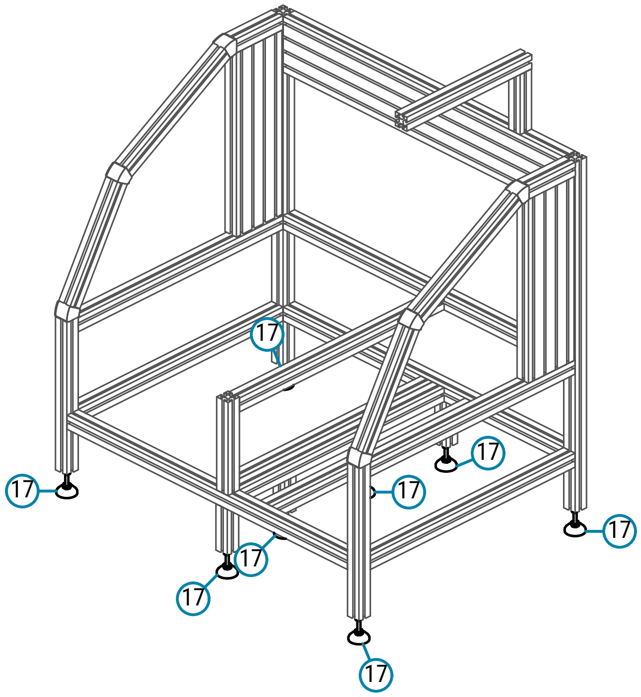

Step 17/ 17

Install 5 x part 15

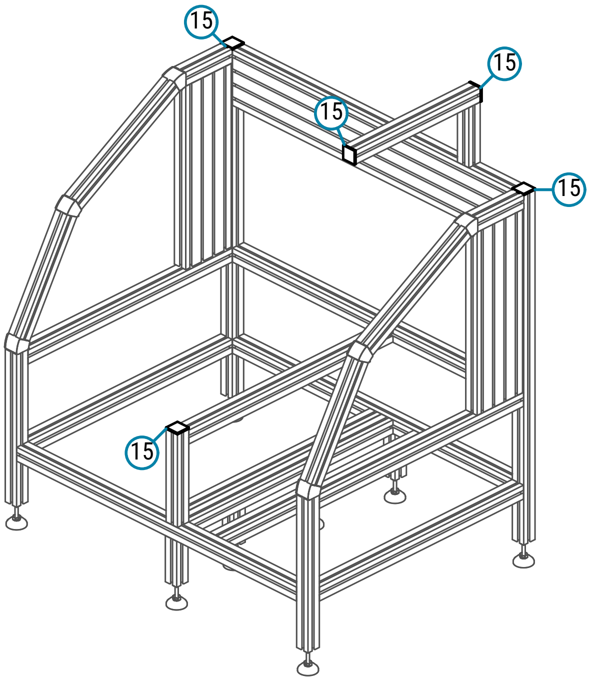

Supplement: Supplementary file 1 [file Data_Sheet_1.pdf]
