# Supplementary material for: Nitrate Feed Improves Growth and Ethanol Production of Clostridium ljungdahlii With CO2 and H2, but Results in Stochastic Inhibition Events
Source: Front Microbiol. 2020 May 6;11:724. doi: 10.3389/fmicb.2020.00724 (PMC7219301; doi:10.3389/fmicb.2020.00724)
Supplement: Supplementary file 2 [file Data_Sheet_2.pdf]

Supplementary Material

Includes:

Supplementary Results S1

Supplementary Figure S1 to S5

Supplementary Table S1

Supplementary Results S1. Concept and assembly of the MBS

All required materials for the MBS (**Supplementary Figure S1**) were purchased from different manufacturers in Germany or France. We provide a list with all information for each unit of the MBS including manufacturer's names, required amounts, and expected costs (**Supplementary Table S1**). The basis for our MBS concept was a commercially available 1-L double-walled glass bottle with GLS 80 neck as the bioreactor vessel. The neck of the bottle was additionally flattened by a glass blower to increase the surface contact area between glass and lid. Furthermore, the custom-made lid was sealed with an additional O-ring. We designed a custom-made lid **(Supplementary Figure S2)**, because it had to offer as many ports as possible to attach pH-regulation and all lines required for a continuous gas and medium feed-in and -out, and we could not find commercially available options that fulfilled our requirements. In addition, the lid had to be autoclavable and gas tight. Our customized lid is made of PTFE and provides vertically attached connection ports for five GL14 fittings and one GL25 fitting. Before attaching the lid, vacuum grease was applied to the glass thread of the bioreactor. One water bath thermostat maintained the temperature of all bioreactors. The equal distribution of the water through black rubber tubing was achieved by installing a starfish manifold. The water outlets of the individual bioreactors were merged into a single line before entering the thermostat again. Stirring bars were used for continuous and unitary agitation of the medium with a multi stirring plate for six bioreactor vessels. Autoclavable pH-electrodes with integrated temperature sensors were installed at the GLS25 port, which was sealed with a PTFE ring that contained a GL25 screw cap. A multi parameter controller, which includes four internal pH/temperature modules, two external pH/temperature modules, and two external relay controllers, was used to track the temperature and to control the pH. All wiring and connections were installed according to the manufacturer's instructions. Twelve peristaltic mini pumps were added to the MBS system and connected to the multi parameter controller to control the pH with base and acid feed for all six bioreactors **(Supplementary Figure S3)**. A three-part stainless-steel tubing set: a sampling tube, an off-gas tube, and an inlet-gas tube with attached micro sparger was designed, custom-built, and attached to each bioreactor. In addition, the sampling tube was extended by a stainless-steel three-way valve. All GLS14 ports of the bioreactor lid were sealed with 3-part GL14 caps containing PTFE/ETFE replacement inner parts. To maintain continuous feed-in and feed-out conditions, a multichannel pump head equipped with twelve channels was attached to a peristaltic pump. For the mini pumps, a chemical-resistant rubber tubing was used. Furthermore, the multichannel pump head was equipped with 2-stop rubber tubing. All rubber parts were connected *via* Luer-Lock adapters and autoclaved *prior* to use. However, the 2-stop rubber tubing was sterilized with bleach (10 vol%) and rinsed with sterile water. To keep the feed bottles anaerobic,

holes for tubing were drilled through GL45 butyl stoppers. Each feed bottle contained three feed lines, which were connected to the 2-stop rubber tubing of the multichannel pump, one tubing line to add sterile vitamins and reducing agents, and one line (made of rubber tubing and a 10-cm piece of a 1-mL glass pipette). For the gas-out line, rubber tubing coming from the bioreactor went first through a 100-mL serum bottle, which was used as water trap before ending in a fermentation airlock for each bioreactor. The effluent of all bioreactors was collected in a single 10-L bottle or six 1-L bottles to track individual effluent levels.

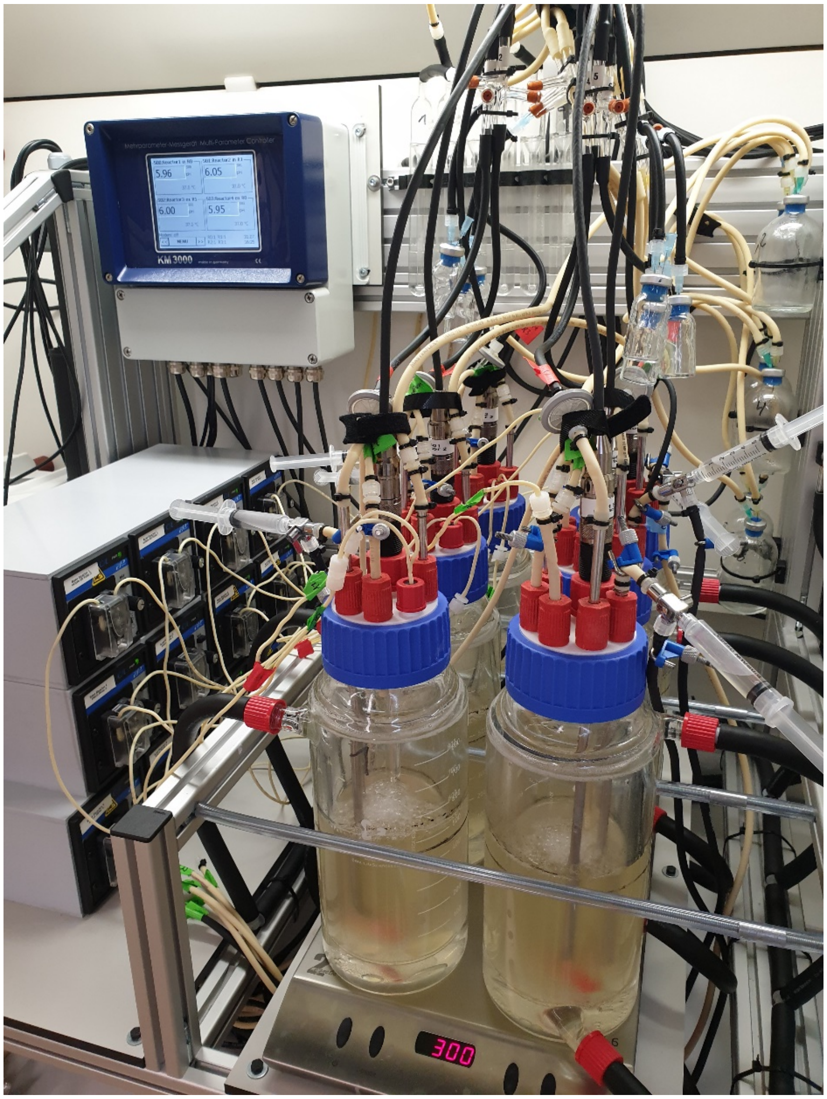

Supplementary Figure S1. The MBS under operating conditions. This picture shows simultaneous growth of *C. ljungdahlii* with CO_2 and H_2 under continuous conditions in six bioreactors with pH-control.

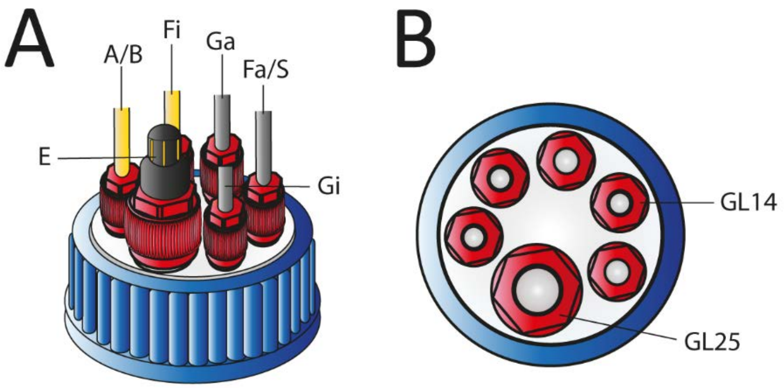

Supplementary Figure S2. Side and top view of the customized lid designed for the MBS. Side view on the lid with connected tubing, ports, and pH-electrode (A). Top view on the lid surface (B). The lid meets all requirements to perform either heterotrophic or autotrophic cultivation experiments with attached pH-control and continuous medium feed-in and -out. Abbreviations: E, pH-electrode; A/B, acid or/and base feed; Fi, medium feed-in line; Ga, gas-out line; Fa/S, medium feed-out line/sampling port *via* three-way valve; Gi, gas inlet, GL14, thread size GL14; GL25, thread size GL25.

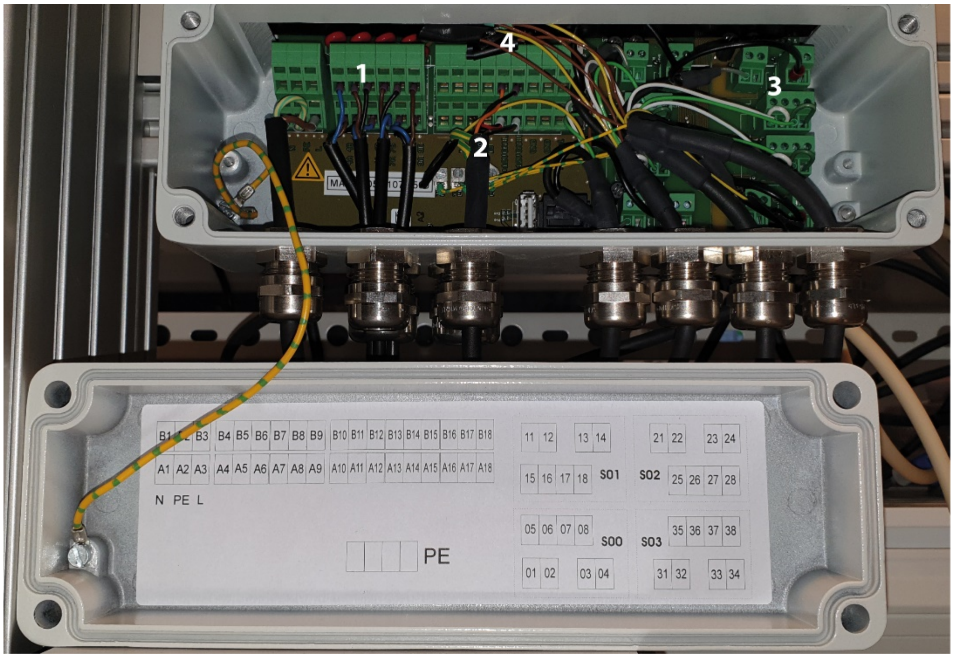

Supplementary Figure S3. Wiring of the multi parameter controller KM3000 to operate six pH-electrodes and twelve pumps *via* relay and CAN bus interface. Four internal relay signals are used to control four pumps directly through 3-wire cables (1). The other eight pumps are controlled as in-line signal *via* CAN bus (2). Four internal interfaces are used to connect four pH/Pt-1000 electrodes (3). It is important to have small bridge connectors for the white and the green wire (not provided with the cable combinations). The other two pH/Pt-1000 electrodes are connected in-line *via* CAN bus. The yellow and brown wire are not required and insulated for safety reasons (4). Unless otherwise stated, all connections were made according to the manufacturer's instructions.

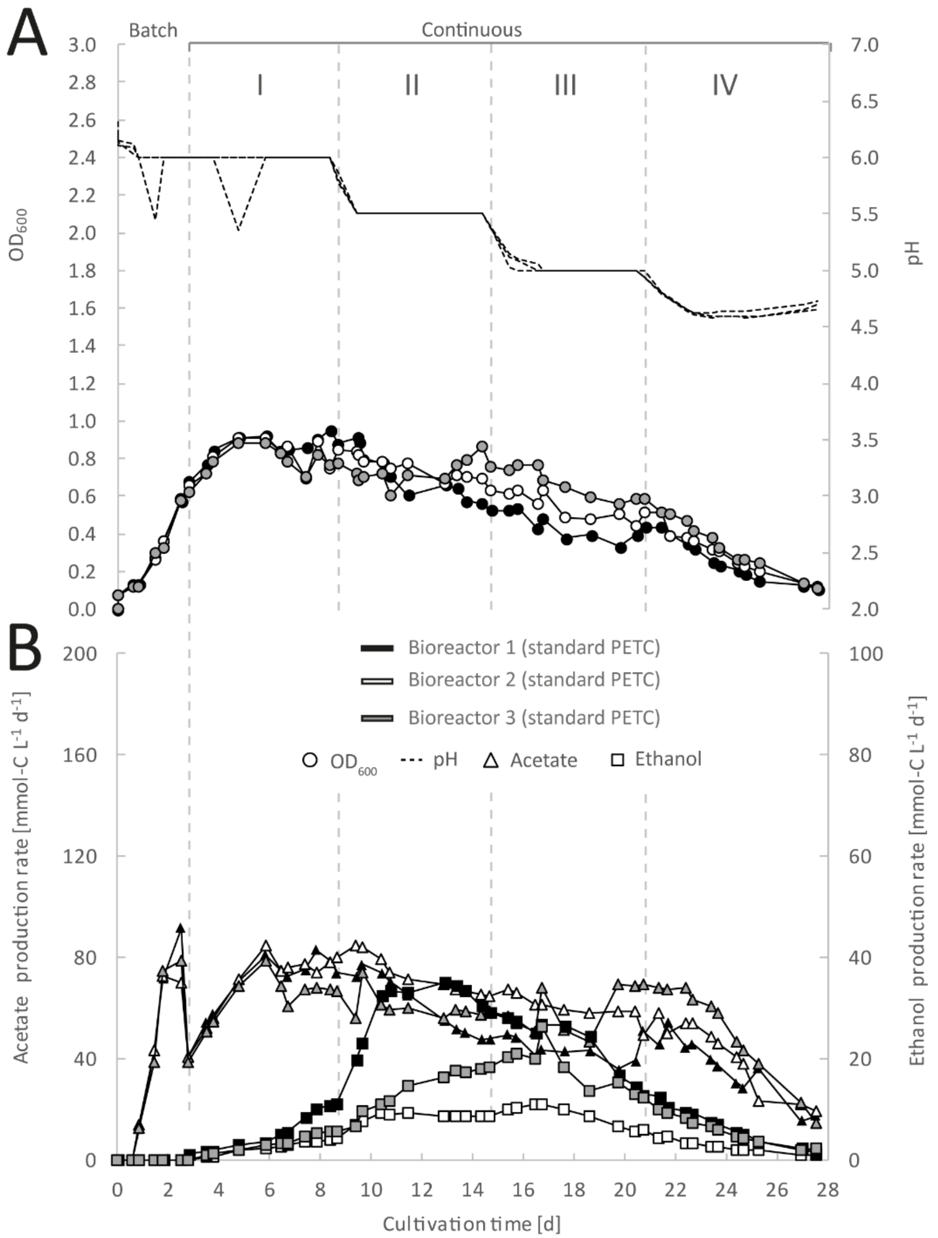

Supplementary Figure S4. Single bioreactor data for continuous gas fermentation of *C. ljungdahlii* with CO_2 and H_2 in standard PETC medium with ammonium at different pH periods. Single values for pH and OD_{600} (A), and for acetate and ethanol production rates in mmol-C L^{-1} d^{-1} (B). The horizontal dotted lines indicate the continuous mode in which medium with different pH was fed to each bioreactor. The pH was not regulated with feeding acid in continuous mode, but by adjusting the feed medium pH to the desired value and by biological acetic acid production. The cultivation volume was initially 500 mL but was tracked daily during the experiment ranging from 500-650 mL. The gas feed rate was 30 mL min^{-1}. The medium feed was 0.10 mL min^{-1} (HRT= ~0.25 d^{-1}). The bioreactors were operated at 37°C and 300 rpm for 27.5 days. pH Period: I, pH=6.0; II, pH=5.5, III, pH=5.0; and IV, pH=4.5.

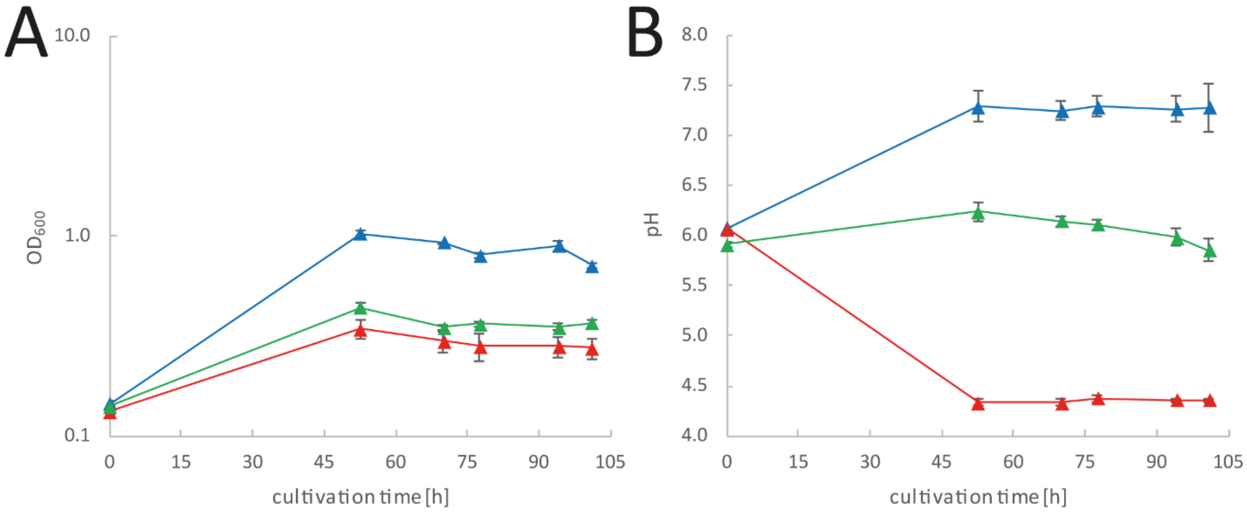

Supplementary Figure S5. Bottle cultivation of *C. ljungdahlii* with CO_2 and H_2 using ammonium, nitrate, or a mixture of both as N-source. Growth as OD_{600} in triplicate (n=3) under each tested condition (A). Changes in pH (B). 50 mL PETC medium supplemented with 0.5 g/L yeast extract but without fructose and ammonium chloride was provided in 240-mL serum bottles. Na-nitrate, ammonium chloride, or a mixture of both N-sources were added as sterile and anaerobic solution. The bottles were filled with sterile CO_2 and H_2 gas (20/80 vol%) to 1 bar overpressure before inoculation. Pre-cultures were grown heterotrophically in PETC with fructose for 48 h at 37°C in a stand incubator, washed once with sterile PBS, concentrated, and directly used for inoculation. Cultivation of main cultures were carried out at 150 rpm in a shaking incubator (Lab companion, ISS-7100R, Jeio Tech, Korea) at 37°C for 101 hours. *C. ljungdahlii* growing with 20 mM Na-nitrate (▲); *C. ljungdahlii* growing with 20 mM ammonium chloride (▲); and *C. ljungdahlii* growing with a mixture of 10 mM Na-nitrate and 10 mM ammonium chloride (▲).

Supplementary Table S1. Required materials for the MBS to operate six bioreactors. The list comprises details about every item and summarizes the total cost of the system (as of Spring, 2019).

**Item**	**Manufacturer**	**Item no.**	**Quantity**	**Approx. cost in €***
1-L double-walled bottle	Duran/VWR	215-4157	6	2000
Customized bioreactor lid	Bohlender/Zinnstag	XZ019-182117	6	2000
O-ring (70x3 mm, FPM30)	Reif	4141280	6	25
Multi stirring plate	2mag	2MAG_10020	1	1250
Set of customized stainless-steel tubing, Ø 6mm, tube (320 mm) with sparger, sample tubing (280 mm), off-gas tubing (100 mm)	bbi-biotech	quote (BZV-2018100900491)	6	2250
Gas manifold for 6 lines (self-built)	Swagelock	-	1	1500
KM3000 Multi-Parameter controller, including 4 internal relays + Can-Bus-Interface (standard version)	Xylem/Si-analytics	90278011	1	2050
Internal pH/temp module	Xylem/Si-analytics	90278011	4	1500
External pH/temp module for KM3000	Xylem/Si-analytics	90278011	2	850
REL 2000 CAN (external module) 4 relays, CAN-Bus-Interface	Xylem/Si-analytics	90278017	2	850
pH-electrode (SL 81-225 pHT VP, pt1000)	Xylem/Si-analytics	90279050	6	2600
Cable combination for SL 81-225 pHT VP	Xylem/Si-analytics	85442000	6	1100
Set of shrink tubing green/yellow (3 mm)	Conrad	541743-62	1	5
Set of wire end ferrule 0.25 mm x 6 mm	Conrad	739539-62	1	5
Masterflex C/L Mini pumps (13 to 80 rpm)	ColeParmer	77122-14	12	9400
Heating thermostat (CC-104A)	Huber	461-1056	1	1600
Octagon-Manifold	Interchim	343938	1	440
Customized bioreactor frame	Item24		1	1200
Plastic 10mm, ESD, grey (300x600mm)	Item24	0.0.614.87	2	15
Plastic 10mm, ESD, grey (300x200mm)	Item24	0.0.614.87	1	5
GL14 cap, 3-parts, including PTFE/ETFE fittings (6 mm)	Bola	D590-06	30	550
GL25 cap with PTFE ring	Bola	H984-03 + H975-18	6	60
Magnetic stirring bar (38 mm)	Carl Roth	A954.1	6	40
Airlock	Chemglass/VWR	AF-0513	6	350
Water trap (240-mL serum bottle)	Glasgerätebau Ochs	102.041	10	20
Masterflex Multichannel pump head + 12 cartridges	ColeParmer	HV-07519-25/SI-07519-85	1	2900
Masterflex L/S pump (100 RPM)	ColeParmer	HV-07528-30	1	1600
2-Stop pump tubing for multichannel pump	ColeParmer	HV-06431-26	1	150
Masterflex mini pump tubing	ColeParmer	GZ-95809-30	6	30
Norprene tubing in size 14	ColeParmer	GZ-06402-14	1	75
Norprene tubing in size 16	ColeParmer	GZ-06402-16	1	75
Masterflex I/P Norprene Tubing A 60 G	ColeParmer	GZ-06404-73	1	50
Luer/Lock fittings in different sizes	Carl Roth	CT59.1-64.1	100	150
3-way valves	Carl Roth	P340.1	6	200
5-L duran bottle	Duran/VWR	215-0057	4	360
10-L duran bottle	Duran/VWR	215-0058	1	150
Butyl stoppers for GL45	Glasgerätebau Ochs	444704	4	15
Tube clamps	Carl Roth	YE70-1	50	100
Zip ties and holder for 6 mm	Hornbach (hardware store)	-	100	30
Screws and nuts	Hornbach (hardware store)	-	20	10
* includes 19% tax			Total cost	**37560**
